# Beyond diffusion: ion and electron migration contribute to charge transport in redox-conducting metal–organic frameworks†

**DOI:** 10.1039/d4sc08246j

**Published:** 2025-02-14

**Authors:** Ben A. Johnson, Ashleigh T. Castner, Hemlata Agarwala, Sascha Ott

**Affiliations:** a Technical University of Munich (TUM), Campus Straubing for Biotechnology and Sustainability Uferstraße 53 Straubing 94315 Germany ben.johnson@tum.de; b Department of Chemistry – Ångström Laboratory, Uppsala University Box 523 75237 Uppsala Sweden Sascha.Ott@kemi.uu.se

## Abstract

Electrical conductivity through redox conducting MOFs (RCMOFs) proceeds by electron hopping between linkers of differing oxidation states. While this process is treated as a purely diffusional process in the literature, we show herein that this prevalent description is an oversimplification, and that emerging electric fields under applied potential result in electron and ion migration which are sizable contributors to charge transport through RCMOFs. This insight is obtained by electrochemical experiments that are conducted under steady-state conditions, which are created by the addition of an electron acceptor to the electrolyte solution, effectively creating a source-drain architecture of the electrode|RCMOF|electrolyte system. In contrast to transient potential-step experiments, such as chronoamperometry that are ubiquitous in the literature, the steady-state conditions in our experiments avoid net ingress or exit of charge balancing counter ions, allowing the assessment of electron diffusion with negligible counter ion flux. The strategy effectively isolates the diffusional response from ion diffusion–migration and electric field effects. Most importantly, it is shown that for transient experiments, the additional flux from migration, resulting from emerging electric fields after the potential step, leads to an overestimation of the experimentally determined apparent diffusion coefficients. The work described herein also demonstrates that the separate determination of electron and ion diffusion through RCMOFs is challenging with simplified models, as the two processes are connected through migration.

## Abstract 2

Restricted ion transport in redox conducting MOFs generates electric fields that enhance electron hopping rates, revealing that charge transport involves mechanisms beyond simple diffusion.

## Graphical abstract

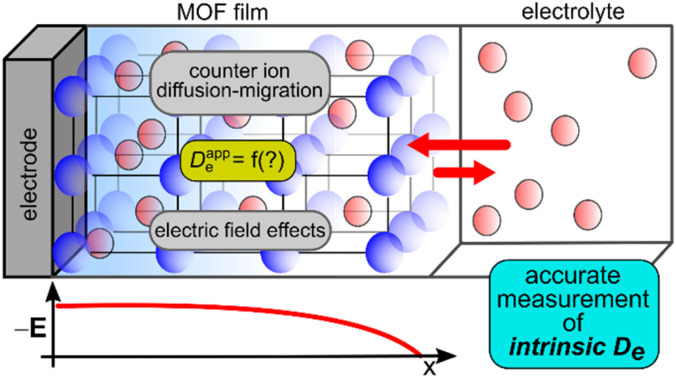

## Introduction

Developing efficient energy conversion and storage systems necessitates materials that exhibit a seemingly contradictory combination of high conductivity and porosity. Metal–organic frameworks (MOFs), composed of a network of metal ions and organic linkers, are a type of porous materials known for their large internal surface areas, crystalline structures, and permanent void spaces.^1,2^ This makes them interesting candidates for electrochemical applications. However, to design MOFs with molecular properties that confer electrical conductivity, a fundamental understanding of the operative charge transport mechanism is essential.

Two distinct conduction mechanisms are possible in MOFs, depending on the degree of orbital overlap and electronic coupling between the molecular components of the framework. In the high coupling limit, charge transport operates by ohmic conduction through a delocalized band structure.^3,4^ On the other hand, when the electronic structure of the MOF is highly localized, *i.e.*, the molecular components have a well-defined standard potential, charge transport takes place by a sequence of outer-sphere electron self-exchange reactions between redox-active linkers or nodes, resulting in a hopping mechanism.^5^ Analogous to redox polymers, MOFs exhibiting this mechanism can be classified as redox conductors (redox-conducting MOFs or RCMOFs), characterized by a measured conductivity that depends on the redox state of the framework, determined by the ratio of oxidized to reduced species within the framework.^6–8^

One major focus in the development of redox-conducting MOFs has been the quantification of electron-hopping rates using planar MOF films.^9–12^ When viewed macroscopically, the ensemble of many individual self-exchange reactions or ‘hops’ resembles a random walk, and charge transport is in fact formally a diffusion process characterized by an equivalent diffusion coefficient *D*_e_.^13,14^ In the simplest case, this macroscopic diffusion coefficient is directly proportional to the microscopic rate of electron self-exchange according to1
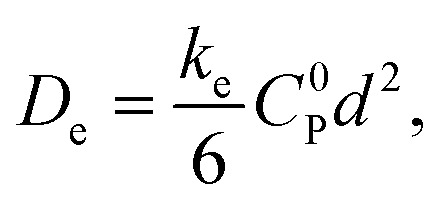
where *k*_e_ (M^−1^ s^−1^) is the bimolecular self-exchange rate constant, *C*^0^_P_ is the total concentration of redox active molecules in the framework, and *d* is the average hopping distance.^15,16^ To experimentally determine an apparent electron-hopping diffusion coefficient, potential-step experiments are widely used,^11,17–28^ where the resulting short-time response is fit to various forms of the Cottrell equation.^29^

While being a simplified model for connecting microscopic electron transfer to macroscopic charge transport, this description—attributing charge transport solely to diffusion from simple electron self-exchange as described by eqn (1)—fails to account for the influence of redox-inactive counter ions, which are introduced as supporting electrolytes and required to maintain electroneutrality within the framework. Consequently, multiple experimental phenomena that demonstrate the coupling of ion mass transport with electron hopping have recently been reported. While the transport rates are dependent on the nature of the electrolyte, previous reports have also explored the effect of ion pairing between the oxidized or reduced species in MOF films and mobile counter ions.^9,10,12,22^ Zooming in further, there are several ways in which the nature of the counter ions (size, concentration, charge *etc.*) can modulate the microscopic electron self-exchange reaction. Electron transfer may occur between ion-paired species, leading to an ion-coupled electron-hopping mechanism.^12^ Analogous to proton-coupled electron transfer, this process can proceed *via* either a stepwise or concerted pathway.^30^ Such studies effectively present a more detailed description of the microscopic self-exchange reactions underlying the electron-hopping mechanism. To provide a complete picture, in addition to microscopic effects, macroscopic mass and charge transfer also need to be considered.

In general, the flux of both electrons and ions includes contributions from: (1) diffusion in response to a concentration (or chemical potential) gradient and (2) migration resulting from any gradient in electrostatic potential across the film.^31^ The latter can be caused either if the ion concentration is deficient with respect to the redox-active components in the MOF or if the rates of ion and electron transport are mismatched, resulting in the build-up of a non-zero electric field in the film. This establishes a potential gradient, resulting in a net flux from migration for both ions and formally electrons. While diffusional processes have been extensively studied in the MOF literature, the role of migration in both ion and electron transport in RCMOFs is frequently overlooked. As a result, the extent to which ion diffusion, electric fields, and migration influence the current response, and ultimately measurements of *D*_e_, remains poorly understood.

**Fig. 1:**
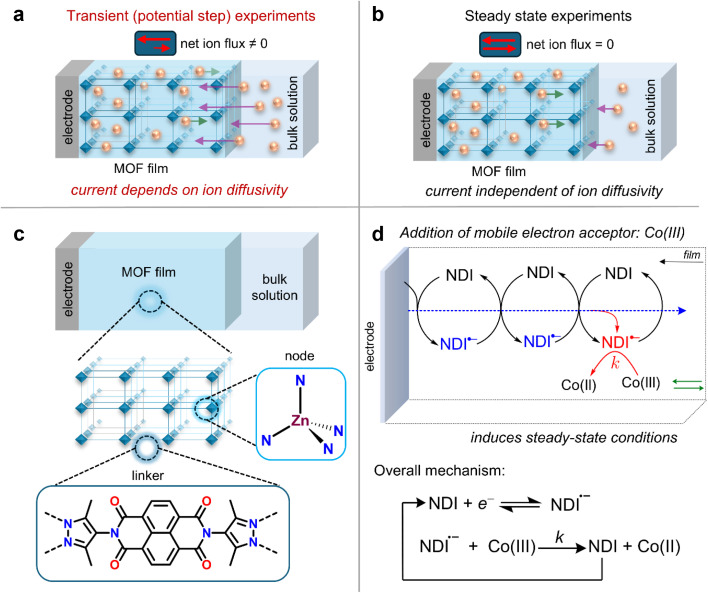
(a) Example of typical transient electrochemical experiments used to measure electron-hopping diffusion coefficients for planar MOF films. This generally consists of monitoring the response of the film after a large potential step (chronoamperometry, chronocoulometry, chronoabsorptometry), followed by application of the Cottrell equation. Such transient methods are characterized by a non-zero net flux for the diffusion–migration of counter ions across the film. The resulting current response is not completely described by the Cottrell equation, and the current is a function of both the counter ion diffusion coefficient *D*_I_ and the electron-hopping diffusion coefficient *D*_e_. (b) A steady-state electrochemical experiment is ideal for extracting *D*_e_ from planar films, where net ion flux is zero, and the current response has minimal interference from *D*_I_. Steady-state is enforced here by mutual competition between electron-hopping diffusion and a chemical reaction (single electron transfer with a freely diffusing acceptor). (c) Schematic structure of the planar Zn(NDI)@FTO MOF films used, showing the chemical structure of the linkers (dipyrazolate naphthalenediimide) and nodes (tetrahedral Zn). (d) Irreversible catalytic-type reaction mechanism involving outer-sphere electron transfer from the reduced linkers in Zn(NDI)@FTO to freely diffusing [Co(bpy)_3_]^3+^ characterized by a second order rate constant *k*.

Currently, most values for electron hopping diffusion that are obtained by transient potential-step measurements of planar films are reported as apparent electron diffusion coefficients *D*^app^_e_. The annotation ‘apparent’ reflects the fact that electron diffusion is affected by the diffusion–migration of counter ions. The transient nature of these measurements entails a non-zero flux of mobile redox-inactive counter ions across the film (Fig. 1a), which is especially evident when the concentration of redox-active species in the MOF greatly exceeds that of the supporting electrolyte (we will show that this is often the case). The unaccounted diffusion–migration of counter ions, which is not considered in the Cottrell equation (*vide infra*), leads to a deviation compared to the intrinsic electron-hopping diffusion coefficient, which we will call *D*_e_ (eqn (1)). This variation arises because the measured response depends on both the diffusion coefficient of the counter ions *D*_I_ and any migration effects due to a potential drop across the film.

Herein, we systematically probe the effects of ion diffusion, electric fields, and migration in RCMOF films by employing experiments designed to operate under an electrochemical steady state. By adding a mobile redox acceptor molecule to the electrolyte, the additional cross reaction between the film and the acceptor mimics a source-drain electrode configuration and generates a steady-state current. This approach was first proposed by Faulkner and co-workers,^32^ who were investigating electron transport through copolymer films containing electrostatically bound redox-active transition metal complexes. At steady-state, the net counter ion flux is zero, and the measured current is independent of the mobile ion diffusivity *D*_I_ (Fig. 1a and b). This can be readily probed by cyclic voltammetry (CV), where we show that the sigmoidal steady-state current–potential response is proportional to *D*_e_.

The migration component of the electron-hopping process will still need to be accounted for if a considerable electric field builds up within the film. This will be the case whenever the total concentration of the redox-active linkers *C*^0^_P_ exceeds that of the mobile counter ions *C*^0^_I_. To reconcile this, we also develop a physico-mathematical model that incorporates field effects due to migration into both the flux of mobile counter ions and electron hopping (formally the migration–diffusion of fixed redox-active linkers). This allows us to more accurately extract *D*_e_ and effectively parse out the relative contributions of ion diffusion and migration to the overall current response. In agreement with previous observations for redox polymers,^33^ our results additionally demonstrate that the overall rate of charge transport in RCMOFs is not governed by a single rate-limiting step, often considered to be either electron hopping or ion diffusion. Instead, these processes occur in parallel, coupled together by migration to maintain electroneutrality, making the concept of a ‘rate-limiting step’ inaccurate in this context.

At this stage, differentiating the intrinsic diffusion coefficient *D*_e_ from an apparent diffusion coefficient *D*^app^_e_ is crucial. The latter is often derived through simple fitting of the Cottrell equation to the transient decay of the current response. This distinction is essential because the assumption that transport is solely governed by semi-infinite electron-hopping diffusion is unlikely to hold for most MOF systems in transient potential step experiments, and ion diffusion–migration plays an important role. Herein, we define the parameter *D*_e_ to only reflect the underlying microscopic electron hopping process. Conversely, we use *D*^app^_e_ to denote a value obtained by a measurement that includes other effects associated with macroscopic transport. This includes the abovementioned counter ion diffusion and electric field effects leading to interference from migration. In other words, *D*^app^_e_ is a function of multiple parameters, for example, *D*^app^_e_ = *f*(*D*_e_, *D*_I_, *C*^0^_I_, *C*^0^_P_, …), where *C*^0^_I_ is the mobile ion concentration, and *C*^0^_P_ is the total electro-active linker concentration in the MOF film.

## Results and discussion

For this study, we chose a previously reported Zn-based MOF with naphthalenediimide (NDI) linkers (Fig. 1c) and 16 Å wide 1D channels (Zn(NDI)@FTO).^34,35^ In our hands, this material forms homogenous thin films on fluorine-doped tin oxide (FTO) substrates (characterized by SEM and PXRD, Fig. S4†) and gives a well-defined electrochemical response,^7,36^ making it an ideal model platform. To create steady-state conditions, a source drain type set-up is created by adding an electron acceptor to the electrolyte. With this set-up, it is possible to monitor the electron transfer reaction from the reduced NDI linkers in the MOF film to the mobile redox acceptor using standard electroanalytical methods. Here, we chose [Co(bpy)_3_]^3+^, which has a formal potential sufficiently positive of that of the NDI linkers to render the mediated reduction of the complex by the film irreversible. The size of [Co(bpy)_3_]^3+^ (approximately 11 Å, obtained from the crystal structure)^37^ is smaller than that of the pores of the MOF.

The overall reaction sequence is displayed in Fig. 1d and matches that of a simple one-step, one-electron catalytic mechanism. The following analysis was performed by varying both the scan rate and the bulk [Co(bpy)_3_]^3+^ concentration for films obtained with thicknesses of 1 μm, as measured by SEM images of film cross sections (Fig. S4b†).

The electrochemical response of the Zn(NDI)@FTO films was first investigated in the absence of [Co(bpy)_3_]^3+^. For comparison, we employed the traditional Cottrell analysis utilizing a large potential step to reduce the NDI linkers of the film (recorded in DMF with 0.5 M LiClO_4_ as the supporting electrolyte). At short time points following this potential step, the time-dependent current response is linearly proportional to *t*^−1/2^. The slope of this linear region was used to calculate *D*^app^_e_ (Fig. S5 and S6†). From this transient chronoamperometry experiment, *D*^app(trans)^_e_ was determined to be 2.5 × 10^−9^ cm^2^ s^−1^. Again, the notation here, *D*^app(trans)^_e_, denotes an apparent diffusion coefficient obtained by a transient electrochemical technique with simple fitting by the Cottrell equation. These are apparent due to the unaccounted influence of counter ion diffusion–migration as well as any electric field effects on electron hopping.

Cyclic voltammograms (CVs) were also recorded in the absence of [Co(bpy)_3_]^3+^, utilizing scan rates spanning more than two orders of magnitude (*ν* = 1 to 500 mV s^−1^) (Fig. S7†). Integration of the current from the cathodic wave at slow scan rates (1 mV s^−1^, Fig. S7a†) allows us to calculate the electro-active surface concentration of NDI linkers as 1 × 10^−7^ mol cm^−2^, which results in a volumetric concentration of approximately *C*^0^_P_ = 1 M.

**Fig. 2:**
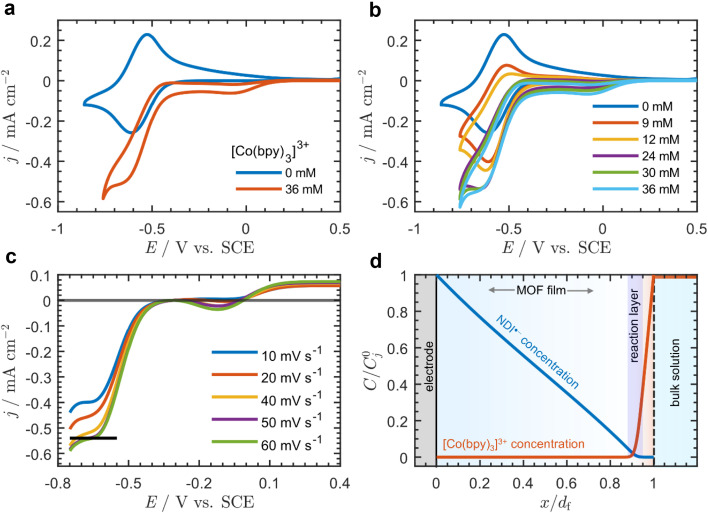
Cyclic voltammograms at 20 mV s^−1^ with 0.5 M LiClO_4_ in DMF of Zn(NDI)@FTO (a) with [Co(bpy)_3_]^3+^ (*C*^0^_A_ = 36 mM) (red) and without (blue) [Co(bpy)_3_]^3+^, and (b) CVs with increasing concentration of [Co(bpy)_3_]^3+^ (*C*^0^_A_ = 0 mM, blue; 9 mM, red; 12 mM, orange; 24 mM, purple; 30 mM, green; 36 mM, cyan). (c) CVs of Zn(NDI)@FTO with *C*^0^_A_ = 36 mM at *ν* = 10 mV s^−1^ (blue), 20 mV s^−1^ (red), 40 mV s^−1^ (orange), 50 mV s^−1^ (purple), and 60 mV s^−1^ (green). The reverse scan was removed for clarity, and the baseline was corrected to account for the residual current from the first wave by translating the CVs such that the current at the foot of the catalytic wave begins at zero (black line shows the quasi-plateau current at *ν* = 50 mV s^−1^). (d) Example dimensionless concentration profiles at the plateau (approximately −0.6 V) showing reduced NDI linkers (blue line) and acceptor (red line, [Co(bpy)_3_]^3+^) as a function of distance normalized to the film thickness (*x* = 0 corresponds to the underlying FTO electrode surface, and *x* = *d*_f_ corresponds to the outer edge of the film at the film-solution interface). Concentration of reduced NDI linkers and acceptor on the vertical axis is normalized to the total concentration of linkers *C*^0^_P_ (*j* = P) and bulk acceptor concentration *C*^0^_A_ (*j* = A) respectively. The thin reaction-layer that develops is highlighted in blue. Concentration profiles were generated from analytical solutions to the physical model presented in the ESI (p. S24),† accounting for diffusion–migration.

Addition of [Co(bpy)_3_]^3+^ to the bulk electrolyte solution results in the appearance of two new waves: one at approximately −0.12 V and another centered on the formal potential of the NDI linkers at −0.56 V *vs.* SCE (*ν* = 20 mV s^−1^) (Fig. 2a). Increasing the bulk concentration of [Co(bpy)_3_]^3+^ (*C*^0^_A_, Fig. 2b) causes an increase in the current at −0.56 V, and the observed wave changes from reversible and peak-shaped to irreversible with a quasi-plateau.

The current continues to increase until *C*^0^_A_ = 24 mM; however, at higher concentrations the plateau current is independent of *C*^0^_A_. This contrasts with the wave near −0.12 V, which is peak shaped for all values of *C*^0^_A_, and the peak current continuously increases with increasing *C*^0^_A_ (Fig. 2b). This behavior as well as background scans on bare FTO in the presence of [Co(bpy)_3_]^3+^ (Fig. S8†) allowed us to assign the first wave at −0.12 V as arising from the direct reduction of [Co(bpy)_3_]^3+^ at the underlying FTO surface. The second wave (−0.56 V) can be assigned to the mediated reduction of [Co(bpy)_3_]^3+^ by the film, given that it exhibits a quasi-sigmoidal response, and the half wave potential is centered near *E*^0^ of the NDI linkers.^16^ Importantly, the Co(iii)/Co(ii) couple (measured on a glassy carbon electrode; Fig. S9†) appears 800 mV positive of the NDI/NDI˙^−^ reduction, resulting in a large driving force for electron transfer, and therefore the cross-reaction between the film and mobile [Co(bpy)_3_]^3+^ species can be considered irreversible.

At high bulk [Co(bpy)_3_]^3+^ concentrations (*C*^0^_A_ ≥ 36 mM) the plateau current from the sigmoidal current–potential response (at scan rates ≥50 mV s^−1^) is approximately scan rate independent (Fig. 2c), indicating that the system is under pure kinetic conditions and there is negligible acceptor depletion at the film-solution interface.^38–40^ As mentioned above, the plateau current is also nearly independent of *C*^0^_A_ at concentrations greater than 24 mM (Fig. 2b).

In the framework of a one-electron catalytic mechanism mediated by a redox film, these two observations uniquely correspond to a situation in which, on the timescale of the reaction, the acceptor only diffuses a very short distance within the film, and the reaction take place in a thin boundary layer near the film-solution interface.^39,41,42^ This boundary layer is so thin, it can be approximated as a surface reaction. Since no reaction is taking place within the bulk of the film, the concentration profile of NDI˙^−^ is almost linear, and the reaction is fast enough to cause the concentration of NDI˙^−^ to drop to zero at the film-solution interface (example concentration profiles are shown in Fig. 2d). The physical meaning of this result is discussed in more detail in Section 3.3.3 of the ESI (p. S8).†

This situation arises from a very fast cross reaction between NDI˙^−^ and [Co(bpy)_3_]^3+^ on the diffusional timescale of both electrons and acceptor. Additionally, the electrons must diffuse further in the film than the acceptor molecule.^39^ With the high concentration of linker in MOFs, these conditions are relatively easy to meet (see ESI, Section 4†). The overall result effectively reproduces the steady-state concentration profiles generated with a source-drain electrode configuration (such as an inter-digitated array electrode).^43^ Here, rather than changing the geometry of the electrode set-up, we are enforcing the desired concentration gradient using a chemical reaction. The underlying electrode surface serves as a source of electrons for the film, while the electron transfer reaction with the added acceptor molecule creates a drain for electrons at the film-solution interface. A derivation demonstrating how this condition generates the desired concentration profiles and steady-state response is presented in the ESI, Section 3.3 (see also Fig. S1).†

Under steady-state conditions induced by the electron transfer reaction with the freely-diffusing acceptor, the current response does not depend on the mobile counter ion diffusivity *D*_I_, as there is no net flux of these counter ions in the film (mathematical justification for this is provided in ESI Section 3.4.1, p. S11†). However, completely analogous to homogenous molecular electrochemistry, an excess concentration of supporting electrolyte compared to that of the analyte molecule is needed to ensure charges are effectively screened in the diffusion layer, resulting in a negligible electrostatic potential gradient, such that there is no contribution from migration to mass transport of the analyte. This is not typically the case for MOF films. Due to their high surface area, the effective concentration of linkers is large, typically on the order of the supporting electrolyte concentration (0.1–1 M). Thus, in contrast to typical solution-phase electrochemical systems, we can expect a significant contribution from migration to both electron-hopping and mass transport of mobile counter ions within the film.

To examine the relative contributions of electron-hopping and migration on charge transport through the framework, we construct a physical model, which allows us to more accurately determine *D*_e_. Our model takes into consideration that while electron-hopping in the absence of an electric field is equivalent to simply diffusion, in the presence of an electrostatic potential gradient, the migration contribution to electron-hopping is not the same as that of an ion. While ion movement is formally monomolecular, electron-hopping is a bimolecular process between discrete redox-active molecules, and the normal Nernst–Planck expression for the flux^31^ does not apply.

This was first enunciated by Savéant,^44^ who derived an appropriate extension to the traditional Nernst–Planck equation, which accommodates the bimolecular nature of electron-hopping. This introduces a second order term in the migration component of the flux. While it has been shown to be significant for analyzing redox materials demonstrating electron-hopping,^45^ this consideration is sometimes omitted in other models.^46^ These models, including ones that have recently gained popularity for investigating charge transport through MOF films,^9,18,47^ may have been derived based on assumptions pertinent to materials other than planar films of redox conductors with discrete sites.^6,7^ We provide a general summary of Poisson–Nernst–Planck theory as it applies to electron-hopping through RCMOF films in the ESI Section 3.1 (pp. S3–S5).† Derivations of analytical expressions for the current–potential response, potential gradient within the film as a function of distance, and ionic concentration profiles are also presented in the ESI Section 3.4–3.6 (pp. S10–S16).† We additionally validated our model by using Marcus theory combined with density functional theory (DFT) calculations to estimate the second order cross-exchange rate constant between the NDI˙^−^ linkers and [Co(bpy)_3_]^3+^ (ESI Section 4 pp. S17–S21†).

**Fig. 3:**
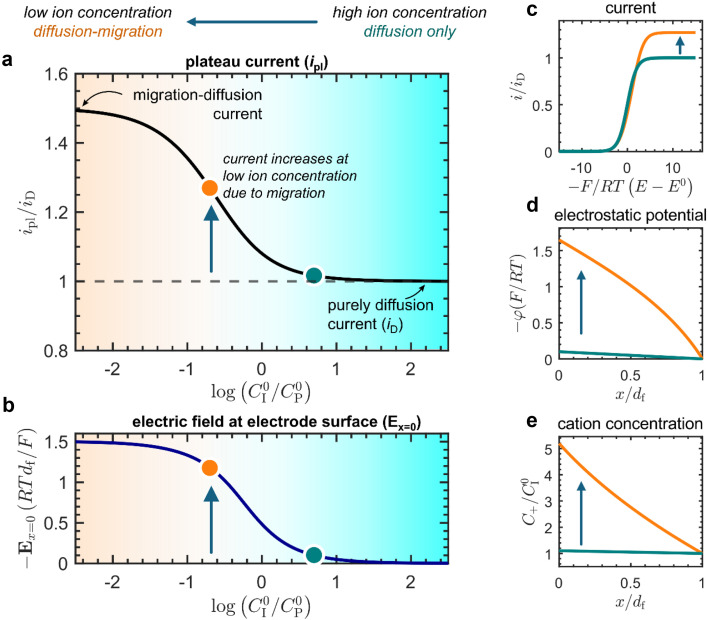
Simulated results from the analytical model (for a summary see ESI p. S24†). (a) Plot of the plateau current (*i*_pl_) *versus* the ratio of mobile ion concentration (*C*^0^_I_) to the total concentration of redox active linkers (*C*^0^_P_). The current is normalized to the current observed in the absence of migration effects (zero electric field) where only diffusion is operative, defined as *i*_D_ = FS*C*^0^_P_*D*_e_/*d*_f_ (ESI, eqn (S44), p. S9†). (b) Corresponding plot of the normalized electric field at the electrode surface as a function of the mobile ion concentration expressed as the ratio *C*^0^_I_/*C*^0^_P_. Two points are highlighted as representative of the purely diffusional response *C*^0^_I_/*C*^0^_P_ = 5 (teal dot) and the ensuing effect of electric fields and transport by migration *C*^0^_I_/*C*^0^_P_ = 0.2 (orange dot). (c) Cyclic voltametric response at two different ion concentrations (*C*^0^_I_/*C*^0^_P_ = 5, teal line; *C*^0^_I_/*C*^0^_P_ = 0.2, orange line), showing the increase in plateau current at low ion concentrations. (d) Electrostatic potential profiles as a function of distance in the film (*C*^0^_I_/*C*^0^_P_ = 5, teal line; *C*^0^_I_/*C*^0^_P_ = 0.2, orange line) plotted at the plateau. (e) Cation concentration profiles as a function of distance in the film (*C*^0^_I_/*C*^0^_P_ = 5, teal line; *C*^0^_I_/*C*^0^_P_ = 0.2, orange line) plotted at the plateau.

The effect of migration on the current response was first analyzed with voltammograms and concentration profiles simulated from the analytical model (Fig. 3). By varying the amount of mobile counter ions (*C*^0^_I_), we found that a low concentration of mobile ions compared with that of the redox-active linkers (*C*^0^_P_) gives rise to an enhancement of the current response. Beginning from excess ion concentration (*C*^0^_I_ ≫ *C*^0^_P_; Fig. 3a, teal dot), as the ratio *C*^0^_I_/*C*^0^_P_ is decreased, the plateau current (*i*_pl_) increases (Fig. 3a, orange dot) up to a maximum of approximately a factor of 1.5 times that of the current observed when only diffusion is operative (*i*_D_). Concurrently, the magnitude of the electric field at the electrode surface (*E*_*x*=0_) also increases with decreasing ion concentration (Fig. 3b). This current enhancement is a direct result of migration affecting both electron hopping and mobile ion diffusion.

Insufficient mobile ion concentration results in an electrostatic potential gradient within the film (Fig. 3d), which induces a corresponding electric field (Fig. 3b). For the system to maintain charge neutrality, the direction of this field will repel anions from and attract cations to the electrode-film interface. Indeed, at steady state, mobile cations are present near the electrode-film interface at higher concentrations than in the bulk electrolyte (Fig. 3e). Furthermore, this means that the dissipative movement of negative charges from migration is in the same direction as the diffusional flux arising from the concentration gradient of reduced linkers (negatively charged species ‘move up’ an electrostatic potential gradient from low to high potential, whereas species tend to ‘move down’ concentration gradients from high to low concentration). Consequently, the net flux of the electron-hopping process is larger than in the absence of the electric field.

A higher current response is then observed in the presence of migration effects (Fig. 3a, orange dot) compared to the case where the transport of both electrons and mobile ions are purely diffusional (Fig. 3a, teal dot). We can observe this as an amplification of the plateau current (*i*_pl_) compared to the purely diffusional current (*i*_D_ = FS*C*^0^_P_*D*_e_/*d*_f_; see ESI eqn (S44), p. S9†) due to migration effects. The limiting current in the CV response increases discernibly as mobile ion concentration decreases below that of the redox active linkers, or when *C*^0^_I_/*C*^0^_P_ ≪ 1 (Fig. 3c). In this regime, the plateau can be described by the following approximate asymptotic expression (see ESI Section 3.5 for derivation†):2
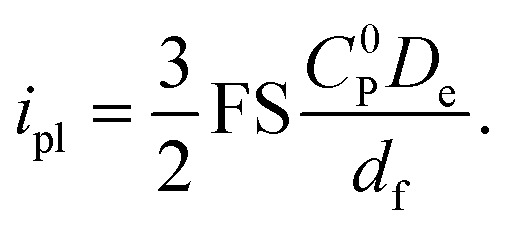


This result corresponds to a 1.5-fold current enhancement due to migration and matches the simulated current in Fig. 3a.

Therefore, the presence of an electric field and ensuing migration resulting from a weakly supported environment in the film (*i.e.*, when *C*^0^_I_/*C*^0^_P_ ≪ 1; Fig. 3, orange dot) will cause measurements of the electron hopping-diffusion coefficient to tend to be overestimated. For example, if an apparent diffusion coefficient *D*^app(ss)^_e_ is extracted from the steady-state plateau current (without considering migration), the application of eqn (2) gives 
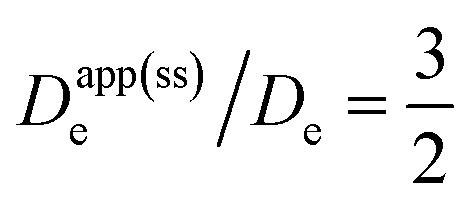
. On the other hand, if the concentration of mobile ions is higher than the total concentration of redox-active linkers, then the system is fully supported (*C*^0^_I_/*C*^0^_P_ ≫ 1) and there is minimal contribution to the current from migration (Fig. 3, teal dot), and *D*^app(ss)^_e_/*D*_e_ = 1. However, this may be difficult to achieve in practice due to the high concentration of linkers (often around 1 M), a characteristic of most MOFs.

**Fig. 4:**
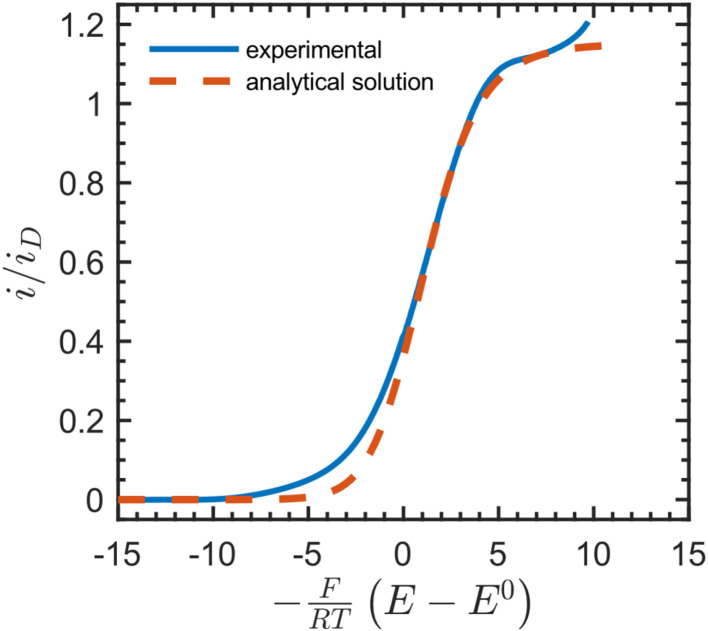
Dimensionless catalytic CV (solid blue line, baseline corrected) in the presence of 36 mM [Co(bpy)_3_]^3+^ at 50 mV s^−1^ in DMF with 0.5 M LiClO_4_, fitted with the analytical solution to the current response (red dotted line) given by eqn (S101)–(S102), ESI.† The plateau current yielded a steady-state value for the electron-hopping diffusion coefficient: *D*_e_ = 5.5 × 10^−10^ cm^2^ s^−1^. Other parameters used as input: *k*^0^_s_ = 1 × 10^−5^ cm s^−1^, *α* = 0.5, *n* = +3, *z* = 0, *C*^0^_P_ = 1 M, *C*^0^_I_ = 0.5 M, *d*_f_ = 1 μm, *E*^0^ = −0.5 V, where *n* and *z* are the charges on the oxidized form of [Co(bpy)_3_]^3+^ and the NDI linkers, respectively. The limiting current when only diffusion is operative (in the absence of migration) is defined as *i*_D_ = FS*C*^0^_P_*D*_e_/*d*_f_.

We then compared the experimental CVs with the current response predicted by the analytical solution (Fig. 4). After baseline subtraction to remove the wave corresponding to direct reduction of the acceptor at the underlying electrode (−0.12 V *vs.* SCE) from the foot of the sigmoidal wave (see Fig. S10†), this resulted in a steady-state measurement for the electron-hopping diffusion coefficient: *D*_e_ = 5.5 × 10^−10^ cm^2^ s^−1^. Best fits resulted from using a formal potential for the NDI linkers of *E*^0^ = −0.5 V *vs.* SCE. This is approximately 70 mV positive of the value obtained by experimental CV in the absence of [Co(bpy)_3_]^3+^ (Fig. 2). We attribute this difference to non-ideal intermolecular interactions between NDI linkers and the diffusing acceptor, or potentially a finite mass transfer rate or partitioning of [Co(bpy)_3_]^3+^ or supporting counter ions at the film-solution interface.^48^

Notably, as shown in the ESI (Section 3.4.1, p. S11),† this value for the electron hopping diffusion coefficient *D*_e_ minimizes the contribution from ion diffusion (independent of *D*_I_) and is corrected for any field effects arising from migration.

Comparing transient techniques employing the Cottrell equation to the steady-state method outlined above, we found that transient chronoamperometry gave a value for the apparent electron diffusion coefficient *D*^app(trans)^_e_ five times larger than the steady-state method, *D*^app(trans)^_e_/*D*_e_ = 5. This indicates that transient experiments are affected to a larger extent by unaccounted migration of both counter ions and electron hopping. Such a result is expected, since under transient conditions, there is an additional source that could contribute to an electrostatic potential gradient in the film. As shown above, a low concentration of ionic species compared to the redox-active linkers will contribute to this effect, but slow ion diffusion is also a possible cause, only present in transient measurements.

This aligns with the theoretical results present in Fig. 3, as well as with previous observations made by Savéant^33^ and Faulkner^32^ for transient experiments conducted on closely related redox polymers. A non-zero potential gradient in the film (whether arising from slow ion movement or a weakly supported system) induces an electric field that acts to enhance the rate of electron hopping, concurrently producing a larger current response. As a result, macroscopic ion transport tends to influence measurements of *D*_e_ in the opposite way as commonly thought: its measured value is larger compared to the case of purely diffusion, rather than reflecting the slower process (electron *vs.* ion diffusion) in the sense of a rate-limiting step.

In fact, the influence of migration on transient potential-step experiments (excluding microscopic effects, ion pairing, *etc.*) will depend on only two dimensionless parameters: the ratio of the intra-MOF ion diffusivity to the intrinsic electron-hopping diffusion coefficient *D*_I_/*D*_e_ and the ratio of the total bulk ion concentration to the total concentration of redox active linkers in the MOF *C*^0^_I_/*C*^0^_P_. If either one of these ratios are small (*D*_I_/*D*_e_ < 1 or *C*^0^_I_/*C*^0^_P_ < 1), an electric field is established as the system attempts to balance the diffusion and migration components of the flux of each charged species. As shown above, a larger current response can be expected, and *D*^app(trans)^_e_, extracted from the slope of the short-time response using the Cottrell equation, will be overestimated (larger than *D*_e_), such that the apparent electron diffusion coefficient is some function of the two dimensionless parameters, *D*^app(trans)^_e_/*D*_e_ = *f*(*D*_I_/*D*_e_, *C*^0^_I_/*C*^0^_P_) where *D*^app(trans)^_e_/*D*_e_ > 1. In contrast, if *D*_I_/*D*_e_ ≫ 1 and *C*^0^_I_/*C*^0^_P_ ≫ 1, the mobile counter ions effectively screen any buildup of electric field in the MOF film, and the obtained value of *D*^app(trans)^_e_ will approach that of the intrinsic *D*_e_. This additionally assumes that the appropriate timescale can be selected where semi-infinite diffusion holds and the diffusion layer thickness is much smaller than the film thickness (*i.e.*, when *t*_exp_ ≪ *d*_f_^2^/*D*_e_, where *t*_exp_ is the experimental timescale and *d*_f_ is the film thickness).

In steady-state experiments, there is zero net flux of ions such that the current response, and therefore *D*^app(ss)^_e_, does not depend on *D*_I_/*D*_e_. We have shown in Fig. 3 (see also eqn (S103) in the ESI, p. S15†) that the steady-state plateau current is only a function of *C*^0^_I_/*C*^0^_P_, such that if a diffusion coefficient is extracted directly from the plateau current, then in general *D*^app(ss)^_e_/*D*_e_ = *f*(*C*^0^_I_/*C*^0^_P_). This can be refined by employing the model we develop here. Upon application of eqn (S103)† to experimental steady-state data (ESI p. S15†), one obtains the intrinsic electron-hopping diffusion coefficient *D*_e_. However, *D*_e_ may still be a function of the nature of the mobile counter ion through microscopic mechanisms such as solvation,^49^ ion pairing, and ion-coupled electron transfer.^12,15,45,50,51^ Microscopic effects are discussed in more detail in the ESI (Section 2, p. S3).†

Summarizing the above discussion, we have addressed challenges related to transient measurements, particularly counterion diffusion and electric field effects (migration). These effects are neither considered in the assumptions underlying the derivation of the Cottrell equation nor experimentally controlled in transient techniques. In such cases, the measured rate of charge transport will include errors linked to unaccounted variables or physical processes, namely the diffusion–migration of charge compensating ions and electric field effects on electron-hopping.

## Conclusion

We find that the introduction of a freely diffusing electron acceptor establishes an electrochemical steady state in RCMOF films, allowing the effects of diffusion and migration of both electron hopping and ion transport to be probed using simple cyclic voltammetry. Two independent measurements of the electron-hopping diffusion coefficient show that steady-state measurements generally give values that more accurately reflect the rate of electron diffusion. Because the measurement is conducted at steady state, this evaluation minimizes any contribution from the diffusivity of the mobile counter ions, since there is no net ionic flux across the film. The corresponding transient measurement resulted in an apparent value for *D*_e_ that was five times larger than the one obtained by the steady-state method.

Using a physico-mathematical model, we were also able to correct for the contribution of migration to electron hopping, which causes an overestimation of measured diffusion coefficients compared to when field effects are absent. When dealing with only macroscopic transport, this corrects the erroneous view that the rate of electron hopping reflects a ‘rate-limiting step,’ often considered to be either electron or ion diffusion. In fact, we have shown that a non-zero electric field within the MOF film increases the flux of electron hopping due to migration, and gives rise to a larger current response. This highlights the need to develop new theoretical models and controlled experimental methods that are specifically tailored to the characteristics of individual experimental systems, beyond simple potential-step methods and the Cottrell model. Similar approaches have been successfully applied in the study of ion insertion into conducting porous materials^52^ as well as gas diffusion or porous electrocatalysts.^53^ This will ultimately lead to an accurate characterization of the conduction properties of redox-conducting MOFs. Finally, the results presented herein have direct implications on future efforts towards synthetic design to improve ion transport and accelerate charge transport in MOFs.

## Data availability

The data supporting this article have been included as part of the ESI.†

## Author contributions

Conceptualization: B. A. J. and S. O.; methodology: B. A. J.; formal analysis: B. A. J. and H. A.; investigation: B. A. J., A. T. C., and H. A.; resources: H. A.; writing – original draft: B. A. J.; writing – review & editing: B. A. J., H. A., and S. O.; visualization: B. A. J. and H. A.; supervision: S. O., B. A. J.; funding acquisition: S. O, H. A.; B. A. J. formulated and solved the physico-mathematical model; H. A. procured and utilized the supercomputing resources and performed DFT studies.

## Conflicts of interest

The authors declare no competing interests.

## Supplementary Material

SC-016-D4SC08246J-s001
