# Supplementary material for: Beyond diffusion: ion and electron migration contribute to charge transport in redox-conducting metal–organic frameworks
Source: Chem Sci. 2025 Feb 14;16(12):5214–22. doi: 10.1039/d4sc08246j (PMC11843255; doi:10.1039/d4sc08246j)
Supplement: SC-016-D4SC08246J-s001 [file SC-016-D4SC08246J-s001.pdf]

Supplementary Information (SI) for Chemical Science.
This journal is © The Royal Society of Chemistry 2025

Supporting Information

Beyond Diffusion: Ion and Electron Migration Contribute to Charge Transport in Redox-Conducting Metal-Organic Frameworks

Ben A. Johnson[1,*], Ashleigh T. Castner[2], Hemlata Agarwala[1], and Sascha Ott[2,*]

[1]Technical University of Munich (TUM), Campus Straubing for Biotechnology and Sustainability, Uferstraße 53, 94315 Straubing, Germany

[2]Department of Chemistry – Ångström Laboratory, Uppsala University Box 523, 75120 Uppsala, Sweden

*Email: ben.johnson@tum.de, Sascha.Ott@kemi.uu.se

Contents

1 Materials and methods **S3**

2 Microscopic effects on electron-hopping diffusion coefficients **S3**

3 Physico-mathematical models **S3**
3.1 Poisson-Nernst-Planck theory for electron-hopping S3
3.1.1 Electromigration of mobile redox-inactive ions in porous media S3
3.1.2 Electron-hopping and electromigration S4
3.1.3 Dynamics of electron-hopping S4
3.2 Steady-state catalytic mechanism S5
3.3 Derivation of steady-state current response in the absence of electromigration . . S6
3.3.1 Dimensionless formulation S6
3.3.2 Perturbation solution S7
3.3.3 Discussion on the location of the reaction layer S8
3.4 Derivation of steady-state current response with electromigration S10
3.4.1 Current response does not depend on mobile counter ion diffusivity at steady-state S11
3.4.2 Dimensionless formulation S11
3.4.3 Analytical solution S12
3.5 Asymptotic approximation for the plateau current S15
3.6 Computational details and model verification S15

4 Model validation with experimental results and DFT calculations **S17**
4.1 Experimental determination of mobile acceptor diffusivity S17
4.2 Estimation of the cross-exchange rate constant S17
4.2.1 Determining a lower bound for k using Damköhler numbers S18
4.2.2 Marcus theory and DFT calculations S18
4.2.3 DFT computational details S20

5 Summary of parameters and symbols **S22**
5.1 Dimensional parameters (Table S1) S22
5.2 Governing dimensionless parameters (Table S2) S23
5.3 Summary of analytical results S24
5.3.1 Diffusion only S24
5.3.2 Including electromigration S24

References **S25**

Figures

S1 Location of the reaction layer S9
S2 Verification of analytical solution including migration S16
S3 Analysis of current response from non-catalytic wave S18
S4 PXRD and SEM images of Zn(NDI)@FTO S25
S5 Chronoamperometry of Zn(NDI)@FTO S26
S6 Cottrell plot of Zn(NDI)@FTO S26
S7 Zn(NDI)@FTO at varying scan rates S27
S8 Background CV of Co(III)$(bpy)_3$ on bare FTO S27
S9 CV of Co(III)$(bpy)_3$ on glassy carbon S28
S10 Background correction applied to catalytic CV S28

1 Materials and methods

All solvents and commercially supplied chemicals were reagent grade and used as received without further purification. Fluorine-doped tin oxide (FTO) substrates (7 Ω/sq) and anhydrous N,N-Dimethylformamide (DMF) (99.8%) were purchased from Sigma-Aldrich. $[Co(bpy)_3](PF_6)_3$ (bpy = 2,2′-bipyridyl) was obtained from TCI Chemicals. Lithium perchlorate ($LiClO_4$) (99+% anhydrous; analysis grade) was purchased from Acros Organics. Zn(NDI)@FTO films were prepared according to a previously reported procedure [1, 2]. Scanning electron microscopy (SEM) images were obtained on a Zeiss LEO 1550 Schottky FEG scanning electron microscope equipped with InLens detector at an acceleration voltage of 3 kV. Film thickness was determined from SEM cross section images using ImageJ [3]. Powder X-ray diffraction patterns (PXRD) were recorded with a Simons D5000 diffractometer (Cu Kα, λ = 0.15418 nm; 45 kV and 40 mA). A parallel beam optics (mirror + mirror) configuration for grazing-incidence XRD measurements was used. Cyclic voltammetry (CV) and chronoamperometry were performed using a one-compartment, three-electrode configuration connected to an Autolab PGSTAT300 potentiostat controlled with GPES 4.9 software (EcoChemie). The electrode set-up included a platinum counter electrode and a saturated calomel electrode (SCE) reference electrode, each separated from the working compartment by a salt bridge. Either bare or modified FTO films (1 cm^2) were used as the working electrode in DMF with 0.5 M $LiClO_4$ as the supporting electrolyte. Ohmic drop was corrected using positive feedback implemented in the instrument. For solution analytes, a glassy carbon (GC) disk working electrode (0.071 cm^2) was used. Zn(NDI)@FTO films were cycled in 0.5 M $LiClO_4$/DMF at 50 mV s^{-1} until the current stabilized before each measurement.

2 Microscopic effects on electron-hopping diffusion coefficients

In addition to macroscopic diffusion-migration of ionic species and of formally electrons, microscopic effects such as intermolecular interactions, ion-pairing, and ion-coupled electron transfer [4–6] can come into play when describing individual electron self-exchange reactions between pairs of linkers [7]. The interpretation of the electron-hopping diffusion coefficient may then deviate from the case of simple electron self exchange. Consequently, such effects will still be present in all measurements even after enforcing conditions that lead to steady-state and after consideration of the macroscopic elecotromigration of counter ions. For the present contribution, we exclude ion-pairing or coupled reactions. For isolated NDI moieties in polar aprotic solvents (DMF), the effect of ion-pairing is significantly diminished [8].

Nonetheless, we emphasize that once the diffusion-migration of counter ions is accounted for in the measurement, even if one or more of the non-ideal effects listed above are still operative, it is the macroscopic diffusion coefficient that is relevant for catalysis, where macroscopic gradients are the driving force for transport, i.e., macroscopic diffusion coefficients will always appear in the the relevant Damköhler number or Thiele modulus for a given diffusion process and chemical reaction. This will hold under the approximation that diffusion coefficients are constants with respect to space and time. Such macroscopic dimensionless governing parameters are important, for example, when evaluating the overall effectiveness of a catalytic film [9].

3 Physico-mathematical models

3.1 Poisson-Nernst-Planck theory for electron-hopping

3.1.1 Electromigration of mobile redox-inactive ions in porous media

The movement of a charged species in porous media, for example an anion or cation through some heterogeneous pore structure permeated by solvent, will occur through two modes of mass

transport: diffusion and electromigration. Consequently the flux in one dimension [10] of a molecular species i, with charge z_i, can be written as

$$\textbf{flux for a mobile ion:}\quad \mathbf{F}_i = -D_i \frac{\partial C_i}{\partial x}\hat{\mathbf{x}} - z_i \frac{F}{RT} D_i C_i \frac{\partial \varphi}{\partial x}\hat{\mathbf{x}}, \tag{S1}$$

where C_i is the concentration of species i, φ is the electric potential, F is Faraday's constant, R is the gas constant, T is temperature, D_i is the molecular diffusion coefficient within the porous material, and $\hat{\mathbf{x}}$ is the unit normal vector pointing perpendicular to the electrode surface. The second term in Eq. S1, formally the migration component of the flux, accounts for the velocity $\mathbf{v}_d$ that a charged molecule experiences resulting from the presence of an electric field:

$$\mathbf{v}_d = z_i \mu_i \mathbf{E}, \tag{S2}$$

where the mobility μ_i of species i is given by the Einstein relation

$$\mu_i = \frac{F}{RT} D_i, \tag{S3}$$

and the electric field $\mathbf{E}$ is the opposite of the gradient in electrostatic potential

$$\mathbf{E} = -\frac{\partial \varphi}{\partial x}\hat{\mathbf{x}}. \tag{S4}$$

3.1.2 Electron-hopping and electromigration

Charge transport through redox-conducting materials [11], such as MOFs with immobilized and discrete molecular redox-active components, occurs through a sequence of outersphere electron self-exchange reactions between adjacent layers in the material. Macroscopically, it is well-known that this conduction mechanism is equivalent to the formal diffusion of the immobile redox-active units [12, 13]. The driving force for this formally diffusional process is a concentration gradient (or more specifically an *electrochemical potential gradient*) created by interfacial charge transfer at an underlying conducting or metallic electrode surface.

Since the redox-active molecules that participate in electron-hopping are charged species (as this involves single electron transfer reactions), electromigration will also come into play in the dynamics of electron-hopping. Savéant illustrated that the bimolecular nature of the underlying microscopic mechanism governing electron-hopping, particularly self-exchange, suggests a parallel second-order behavior for the migration component of the flux [14]. Consequently, he demonstrated that the flux of an immobile redox-active species j in one dimension can be expressed as

$$\textbf{flux for electron-hopping:}\quad \mathbf{F}_j = -D_j \frac{\partial C_j}{\partial x}\hat{\mathbf{x}} - z_j \frac{F}{RT} D_j C_j \left(1 - \frac{C_j}{C_j^0}\right) \frac{\partial \varphi}{\partial x}\hat{\mathbf{x}}, \tag{S5}$$

where D_j is the electron-hopping diffusion coefficient, z_j is the charge on j, and C_j^0 is the total concentration of immobile redox active molecules in the material.

3.1.3 Dynamics of electron-hopping

In a typical experiment, the applied potential will trigger charge transfer at the underlying electrode surface. This perturbs the surface concentration of the immobile redox-active species in the first layer of the film, creating a concentration gradient that initiates the electron-hopping process. This is accompanied by transport of mobile redox-inactive counter ions (generally composing the supporting electrolyte) as the system tries to maintain charge neutrality. It is the coupling between these two processes (diffusional charge transport and mass transport of

mobile charge-compensating ions) that gives rise to electric field effects and migration in the context of electron-hopping.

By applying mass conservation to Eqs. S1 and S5, we can derive the spatiotemporal evolution of the concentration for each redox-active species and the mobile counter ions. This outcome is commonly known as the Nernst-Planck equation.

The Nernst-Planck equation for a mobile redox-inactive counterion i is

$$\textbf{mobile ions:} \quad \frac{\partial C_i}{\partial t} = D_i \frac{\partial^2 C_i}{\partial x^2} + z_i \frac{F}{RT} D_i \frac{\partial}{\partial x}\left(C_i \frac{\partial \varphi}{\partial x}\right). \tag{S6}$$

Nernst-Planck equation for a immobile redox species j engaged in electron hopping is

$$\textbf{electron-hopping:} \quad \frac{\partial C_j}{\partial t} = D_j \frac{\partial^2 C_j}{\partial x^2} + z_j \frac{F}{RT} D_j \frac{\partial}{\partial x}\left[C_j \left(1 - \frac{C_j}{C_j^0}\right) \frac{\partial \varphi}{\partial x}\right]. \tag{S7}$$

This system is closed using Poisson's equation, which describes the electricstatic potential distribution resulting from a given charge density:

$$\textbf{Poisson's equation:} \quad \frac{\partial^2 \varphi}{\partial x^2} = -\frac{\rho}{\varepsilon_r \varepsilon_0}, \tag{S8}$$

where ε_r is the relative permittivity of the medium, ε_0 is the permittivity of vacuum and ρ is the charge density given by

$$\rho = F \sum_k z_k C_k. \tag{S9}$$

The system of partial differential equations comprising Eqs. S6, S7, and S8 are generally referred to as the ***Poisson-Nernst-Planck equations*** [10], detailed here specifically for the case of one-dimensional electron hopping in redox-conducting materials [14].

3.2 Steady-state catalytic mechanism

$$\mathrm{P}^z + e^- \leftrightharpoons \mathrm{Q}^{z-1} \qquad \{E^0,\ k_s^0,\ \alpha\}$$

$$\mathrm{Q}^{z-1} + \mathrm{A}^n \rightarrow \mathrm{P}^z + \mathrm{B}^{n-1} \qquad \{k\}$$

Here we consider the reaction diffusion problem for a 1D planar film depicted in Figure 1c-d in the main text. In this formulation, P is the oxidized form of the linker (NDI), Q is the reduced form of the linker ($NDI^{\bullet -}$), the acceptor molecule A is nominally the substrate of the cross reaction ($[Co(bpy)_3]^{3+}$), and B is the product ($[Co(bpy)_3]^{2+}$). This supplements the results for Zone E + S from Costentin and Savéant [15, 16]; however, the following derivation additionally includes the possibility of slow electron transfer at the electrode-film interface. In the following section, we then formally account for the diffusion-migration of both the immobile redox-active linkers and redox-inactive mobile counter ions within the film in addition to the catalytic reaction.

Outer sphere electron transfer at the electrode-film interface ($x = 0$) generates the reduced linker Q, which propagates away from the electrode via electron-hopping diffusion, described by an equivalent diffusion coefficient D_e. The electrochemical reaction at the interface is modeled by Butler–Erdey-Grúz–Volmer kinetics [17, 18]. Within the film of thickness d_f, the reduced linker Q participates in a cross reaction with a freely diffusing acceptor (substrate) A, characterized by a second order rate constant k. The diffusivity of the acceptor A within the film is given by D_A. In the scenario being examined, the cross reaction between these two species is very fast compared to their respective diffusion times. Since sigmoidal, S-shaped voltammograms were obtained, the reaction-diffusion processes inside the film are at steady-state on the voltammetric timescale. Additionally, this implies that the acceptor concentration at the film-solution interface ($x = d_\mathrm{f}$) is maintained at its bulk value throughout the scan.

3.3 Derivation of steady-state current response in the absence of electromigration

For the following derivation, we take the potential drop within the film to be zero. The particular case that includes the formal migration-diffusion of the fixed linkers as well as the cross reaction in the presence of acceptor (substrate) will be discussed in the next section (3.4).

The steady-state governing equations ($0 < x < d_\mathrm{f}$) can be written as

$$D_\mathrm{e}\frac{d^2Q}{dx^2} - kQA = 0, \tag{S10}$$

$$D_\mathrm{A}\frac{d^2A}{dx^2} - kQA = 0, \tag{S11}$$

with the boundary conditions given by

$$Q(0) = Q_0, \quad \frac{dQ}{dx}(d_\mathrm{f}) = 0, \tag{S12}$$

$$A(d_\mathrm{f}) = C_\mathrm{A}^0, \quad \frac{dA}{dx}(0) = 0, \tag{S13}$$

where Q_0 is the concentration of species Q at the electrode surface, and C_A^0 is the bulk acceptor (A) concentration. The concentrations of P and Q are conserved scalar quantities such that

$$P(x) + Q(x) = C_\mathrm{P}^0. \tag{S14}$$

The current is defined as

$$i = -FSD_\mathrm{e}\frac{dQ}{dx}(0). \tag{S15}$$

From this point, we adopt the (arbitrary) convention that cathodic current is defined as positive for convenience. The electrode kinetics are described by the Butler–Erdey-Grúz–Volmer equation [17, 18]

$$i = FSk_s^0 e^{-\frac{\alpha F}{RT}(E-E^0)}\left(P(0) - Q(0)e^{\frac{F}{RT}(E-E^0)}\right), \tag{S16}$$

where F is Faraday's constant, S is the surface area of the electrode, E^0 is the standard potential of the P/Q couple, α is the transfer coefficient, and k_s^0 is the standard rate constant for interfacial electron transfer.

3.3.1 Dimensionless formulation

We introduce the following dimensionless variables:

$$p = \frac{P}{C_\mathrm{P}^0}, \quad q = \frac{Q}{C_\mathrm{P}^0}, \quad a = \frac{A}{C_\mathrm{A}^0}, \quad y = \frac{x}{d_\mathrm{f}}, \quad \psi = \frac{i}{FSD_\mathrm{e}\frac{C_\mathrm{P}^0}{d_\mathrm{f}}}, \quad \xi = -\frac{F}{RT}\left(E - E^0\right),$$

which results in the system of dimensionless equations:

$$\frac{d^2q}{dy^2} - \mathrm{Da}_\mathrm{e}qa = 0, \tag{S17}$$

$$\frac{d^2a}{dy^2} - \mathrm{Da}_\mathrm{A}qa = 0, \tag{S18}$$

$$q(0) = q_0, \quad \frac{dq}{dy}(1) = 0, \tag{S19}$$

$$a(1) = 1, \quad \frac{da}{dy}(0) = 0, \tag{S20}$$

$$\psi = -\frac{dq}{dy}(0) = \Lambda e^{\alpha\xi}\left(1 - q_0\left(1 + e^{-\xi}\right)\right). \tag{S21}$$

The three governing dimensionless parameters are

$$\mathrm{Da_e} = d_\mathrm{f}^2 \frac{kC_\mathrm{A}^0}{D_\mathrm{e}}, \qquad \mathrm{Da_A} = d_\mathrm{f}^2 \frac{kC_\mathrm{P}^0}{D_\mathrm{A}}, \qquad \Lambda = \frac{d_\mathrm{f} k_s^0}{D_\mathrm{e}}. \tag{S22}$$

Dimensionless cathodic current is defined as positive for convenience.

3.3.2 Perturbation solution

Fast reactions on the diffusion timescale of both the freely diffusing acceptor and electron-hopping implies that $\mathrm{Da_e} \gg 1$, $\mathrm{Da_A} \gg 1$ (this is the condition that yields the behavior described previously [15]). Therefore, we can define two small parameters in the problem. Let $\epsilon_1 := \mathrm{Da_e^{-1}}$ and $\epsilon_2 := \mathrm{Da_A^{-1}}$ such that $0 < \epsilon_1, \epsilon_2 \ll 1$. This gives

$$\epsilon_1 \frac{d^2 q}{dy^2} - qa = 0, \tag{S23}$$

$$\epsilon_2 \frac{d^2 a}{dy^2} - qa = 0. \tag{S24}$$

We now expand q and a in a regular perturbation series [19, 20]

$$q \sim q_{0,0} + \epsilon_1 q_{1,0} + \epsilon_2 q_{0,1} + \epsilon_1\epsilon_2 q_{1,1} + ..., \tag{S25}$$

$$a \sim a_{0,0} + \epsilon_1 a_{1,0} + \epsilon_2 a_{0,1} + \epsilon_1\epsilon_2 a_{1,1} + ..., \tag{S26}$$

and substitute these into Eqs. S23 and S24. Collecting terms with like powers yields

$$\epsilon_1 \frac{d^2 q_{0,0}}{dy^2} + \epsilon_1^2 \frac{d^2 q_{1,0}}{dy^2} + \epsilon_2\epsilon_1 \frac{d^2 q_{0,1}}{dy^2} + ... - (q_{0,0}a_{0,0} + \epsilon_1 q_{0,0}s_{1,0} + \epsilon_1 s_{0,0}q_{1,0} + ...) = 0, \tag{S27}$$

$$\epsilon_2 \frac{d^2 a_{0,0}}{dy^2} + \epsilon_1\epsilon_2 \frac{d^2 a_{1,0}}{dy^2} + \epsilon_2^2\epsilon_1 \frac{d^2 a_{0,1}}{dy^2} + ... - (q_{0,0}a_{0,0} + \epsilon_1 q_{0,0}a_{1,0} + \epsilon_1 a_{0,0}q_{1,0} + ...) = 0. \tag{S28}$$

At leading order $\mathcal{O}(1)$ we find

$$q_{0,0}a_{0,0} = 0. \tag{S29}$$

This equation must satisfy both boundary conditions for a and q at each end of the film. This suggests that the film can be divided into two outer regions: one on the left hand side corresponding to the Dirichlet boundary condition at the electrode-film interface, and one on the right hand side corresponding to the Dirichlet boundary condition at the film-solution interface. Applying these to Eq. S29 in the appropriate region yields

In the left outer region:

$$q_{0,0}(0) = q_0 \qquad \Rightarrow \qquad a_{0,0}^L \equiv 0. \tag{S30}$$

In the right outer region:

$$a_{0,0}(1) = 1 \qquad \Rightarrow \qquad q_{0,0}^R \equiv 0. \tag{S31}$$

This result may seem trivial, but this leads us to the key characteristic of Zone E + S [15]: the reaction is fast compared to both diffusion timescales ($d_\mathrm{f}^2/D_\mathrm{e}$, $d_\mathrm{f}^2/D_\mathrm{A}$), such that Q and A cannot coexist together in the outer regions [21], where Q and A simply exhibit approximately steady-state diffusion (linear concentration profiles). Instead, the reaction is confined to a thin boundary layer somewhere in the *interior* of the film rather than directly adjacent to an interface.

This is commonly referred to as an *interior layer* in the context of boundary layer theory [19, 20].

We proceed by subtracting Eq. S23 from Eq. S24, which gives

$$\frac{d^2q}{dy^2} - \eta\frac{d^2a}{dy^2} = 0, \tag{S32}$$

with η defined as

$$\eta = \frac{\epsilon_2}{\epsilon_1} = \frac{D_{\mathrm{A}}C_{\mathrm{A}}^0}{D_{\mathrm{e}}C_{\mathrm{P}}^0}. \tag{S33}$$

Integration yields

$$q - \eta a = c_1 y + c_2. \tag{S34}$$

Applying the results from the two outer regions allows us to evaluate the constants of integration:

$$q(0) = q_0,\ a_{0,0}^L \equiv 0 \qquad \Rightarrow \qquad c_2 = q_0, \tag{S35}$$

$$a(1) = 1,\ q_{0,0}^R \equiv 0 \qquad \Rightarrow \qquad c_1 = -(\eta + q_0). \tag{S36}$$

This gives the concentration profiles in the outer layers:

$$q \sim q_{0,0}^L = -\left(\eta + q_0\right) y + q_0, \tag{S37}$$

$$a \sim a_{0,0}^R = \left(\frac{\eta + q_0}{\eta}\right) y - \eta^{-1} q_0. \tag{S38}$$

These linear profiles are accurate to $\mathcal{O}(\mathrm{Da}_{\mathrm{e}}^{-1}, \mathrm{Da}_{\mathrm{A}}^{-1})$. Differentiating Eq. S37 and evaluating at $y = 0$ gives

$$\frac{dq}{dy}(0) = -\left(\eta + q_0\right). \tag{S39}$$

Using the definition of the current (Eq. S21) we find

$$q_0 = \psi - \eta. \tag{S40}$$

Eliminating q_0 from Eq. S21 finally results in

$$\psi = \frac{1}{1 + \frac{1}{\Lambda}e^{-\alpha\xi} + e^{-\xi}} + \frac{\eta\left(1 + e^{-\xi}\right)}{1 + \frac{1}{\Lambda}e^{-\alpha\xi} + e^{-\xi}}. \tag{S41}$$

3.3.3 Discussion on the location of the reaction layer

The parameter η essentially controls the location of the thin reaction layer within the film (Figure S1), depending on the balance of electron hopping diffusion and acceptor diffusion as well as the total concentration of reduced catalyst and substrate (acceptor) at either end of the film. When $0 < \eta^{-1} \ll 1$, the acceptor diffuses further than the electrons, and the reaction plane lies next to the electrode-film interface (Figure S1a). As such, the current reflects the rate of acceptor diffusion. Conversely, when $0 < \eta \ll 1$, the reaction plane is located close to the film-solution interface. Now, the catalytic reaction acts as a drain for electrons at the outermost edge of the film (Figure S1c). In the present case, due to the large concentration of linkers (P/Q), $D_{\mathrm{A}}C_{\mathrm{A}}^0 \ll D_{\mathrm{e}}C_{\mathrm{P}}^0$, which means $\eta \to 0$. In this limit, the current is controlled only by electron-hopping diffusion through the film in addition to interfacial electron transfer governed by Λ. We arrive at the leading order approximation for small η given by

$$\psi = \frac{1}{1 + \frac{1}{\Lambda}e^{-\alpha\xi} + e^{-\xi}}. \tag{S42}$$

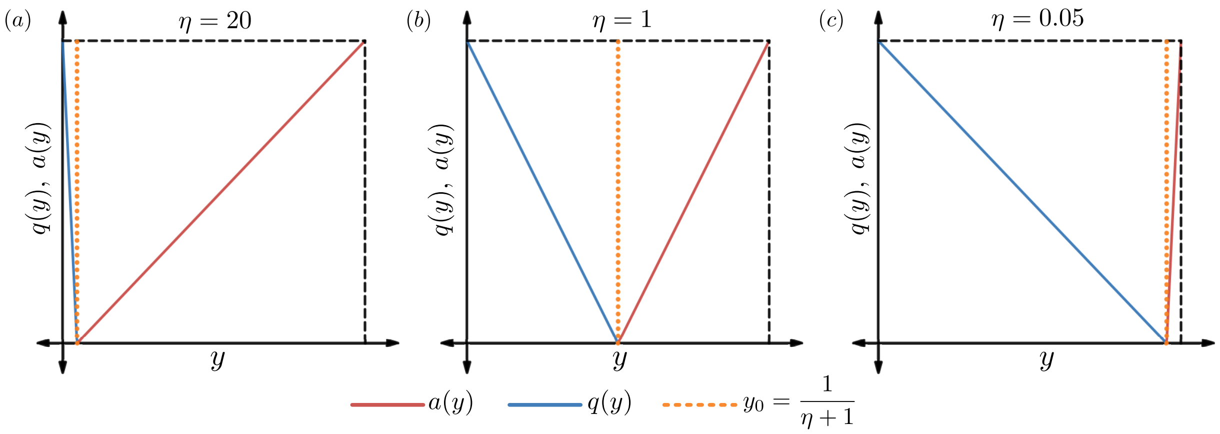

Figure S1. Concentration profiles of reduced catalyst q (blue line) and acceptor a (red line) as a function of distance within the film $0 \leq y \leq 1$. These are plotted from Eqs. S37 and S38 using the boundary condition $q_0 = 1$ with different values of η. The leading order approximation for the plane that describes the location of the reaction layer is given by y_0 (orange dotted line).

Back in dimensional form we have

$$i = \frac{FSC_{\mathrm{P}}^0 \frac{D_{\mathrm{e}}}{d_{\mathrm{f}}}}{1 + \left(\frac{D_{\mathrm{e}}}{d_{\mathrm{f}} k_s^0}\right) e^{\left[\frac{\alpha F}{RT}(E - E^0)\right]} + e^{\left[\frac{F}{RT}(E - E^0)\right]}}, \tag{S43}$$

and the plateau current is given by

$$i_{\mathrm{pl}} = FS\frac{C_{\mathrm{P}}^0 D_{\mathrm{e}}}{d_{\mathrm{f}}}. \tag{S44}$$

We can define this as the diffusion current in the absence of migration: $i_{\mathrm{D}} := FSC_{\mathrm{P}}^0 D_{\mathrm{e}}/d_{\mathrm{f}}$.

3.4 Derivation of steady-state current response with electromigration

We now will analyze the same catalytic reaction as above; however, now adding the diffusion-migration of mobile redox inactive ionic species. The charge is compensated by a neutral binary supporting electrolyte composed of freely diffusing cation, U^+, and anion, V^-, at a bulk concentration given by C_I^0. We employ the Poisson-Nernst-Planck (PNP) equations as the suitable theoretical framework. Additionally, we incorporate a correction to the migration term, which addresses electron-hopping between immobile linkers, as proposed by Savéant [14]. This correction modifies the Nernst-Planck equation by considering the bimolecular nature of electron-hopping within the migration term of the flux as described above. At steady-state the governing equations are

$$D_e \frac{d^2P}{dx^2} + z\frac{F}{RT}D_e \frac{d}{dx}\left(P\left(1-\frac{P}{C_P^0}\right)\frac{d\varphi}{dx}\right) + kQA = 0, \tag{S45}$$

$$D_e \frac{d^2Q}{dx^2} + (z-1)\frac{F}{RT}D_e \frac{d}{dx}\left(Q\left(1-\frac{Q}{C_P^0}\right)\frac{d\varphi}{dx}\right) - kQA = 0, \tag{S46}$$

$$D_A \frac{d^2A}{dx^2} + n\frac{F}{RT}D_A \frac{d}{dx}\left(A\frac{d\varphi}{dx}\right) - kQA = 0, \tag{S47}$$

$$D_A \frac{d^2B}{dx^2} + (n-1)\frac{F}{RT}D_A \frac{d}{dx}\left(B\frac{d\varphi}{dx}\right) + kQA = 0, \tag{S48}$$

$$D_+ \frac{d^2U}{dx^2} + \frac{F}{RT}D_+ \frac{d}{dx}\left(U\frac{d\varphi}{dx}\right) = 0, \tag{S49}$$

$$D_- \frac{d^2V}{dx^2} - \frac{F}{RT}D_- \frac{d}{dx}\left(V\frac{d\varphi}{dx}\right) = 0, \tag{S50}$$

where U and V are the monovalent mobile counter cation and anion concentrations with intra-MOF diffusivities D_+ and D_- respectively, C_P^0 is the total concentration of redox-active linkers, φ is the electric potential, F is Faraday's constant, R is the gas constant, T is temperature, and z and n are the charges on the oxidized form of the linker (P) and freely diffusing acceptor (A) respectively. Poisson's equation describes the electric potential distribution:

$$\frac{d^2\varphi}{dx^2} = -\frac{\rho}{\varepsilon_r\varepsilon_0}, \tag{S51}$$

where ε_r is the relative permittivity of the medium, ε_0 is the permittivity of vacuum and ρ is the charge density given by

$$\rho = F\left(zP + (z-1)Q + nA + (n-1)B + U - V\right). \tag{S52}$$

The steady-state current is defined as

$$\frac{i}{FS} = -D_e\left(\frac{dQ}{dx}(0) + (z-1)\frac{F}{RT}Q(0)\left(1-\frac{Q(0)}{C_P^0}\right)\frac{d\varphi}{dx}(0)\right). \tag{S53}$$

with cathodic current taken as positive for convenience. The electrode kinetics are given by

$$i = FSk_s^0 e^{-\frac{\alpha F}{RT}(E-E^0-\varphi_0)}\left(P(0) - Q(0)e^{\frac{F}{RT}(E-E^0-\varphi_0)}\right). \tag{S54}$$

Compared to Eq. S16, here we have modified the exponential terms to account for any loss in driving force due to the electric field that can develop within the film [22]. The electrostatic potential at the edge of the double layer in our model corresponds to $x = 0$, and we adopt the notation $\varphi(x=0) = \varphi_0$, using the electroneutral bulk solution as a reference point: $\lim_{x\to\infty} \varphi = 0$.

3.4.1 Current response does not depend on mobile counter ion diffusivity at steady-state

At this point it is important to note that from straightforward examination of Eqs. S49 and S50, the diffusivities of the mobile counterions within the MOF film, denoted as D_+ and D_-, cancel out of the governing equations. Consequently, *the current response is independent of the ionic diffusion coefficients under steady-state conditions*:

$$\frac{d}{dx}\left(\frac{dU}{dx}+\frac{F}{RT}U\frac{d\varphi}{dx}\right)=0, \tag{S55}$$

$$\frac{d}{dx}\left(\frac{dV}{dx}-\frac{F}{RT}V\frac{d\varphi}{dx}\right)=0. \tag{S56}$$

Simple integration and application of the relevant boundary conditions also *demonstrates that the net fluxes of the mobile counter cation and anion in the film are identically zero whenever steady-state holds*:

$$F_{\mathrm{U}}=D_+\left(\frac{dU}{dx}+\frac{F}{RT}U\frac{d\varphi}{dx}\right)=0, \tag{S57}$$

$$F_{\mathrm{V}}=D_-\left(\frac{dV}{dx}-\frac{F}{RT}V\frac{d\varphi}{dx}\right)=0. \tag{S58}$$

3.4.2 Dimensionless formulation

We now introduce the following dimensionless variables

$$p=\frac{P}{C^0_{\mathrm{P}}},\qquad q=\frac{Q}{C^0_{\mathrm{P}}},\qquad a=\frac{A}{C^0_{\mathrm{A}}},\qquad b=\frac{B}{C^0_{\mathrm{A}}},\qquad y=\frac{x}{d_{\mathrm{f}}},\qquad u=\frac{U}{C^0_{\mathrm{I}}},\qquad v=\frac{V}{C^0_{\mathrm{I}}},$$

$$\psi=\frac{i}{FSD_e\frac{C^0_{\mathrm{P}}}{d_{\mathrm{f}}}},\qquad \xi=-\frac{F}{RT}\left(E-E^0\right),\qquad \phi=\frac{\varphi}{RT/F}.$$

This results in the following dimensionless equations:

$$\frac{d^2p}{dy^2}+z\frac{d}{dy}\left(p\left(1-p\right)\frac{d\phi}{dy}\right)+\mathrm{Da}_{\mathrm{e}}qa=0, \tag{S59}$$

$$\frac{d^2q}{dy^2}+(z-1)\frac{d}{dy}\left(q\left(1-q\right)\frac{d\phi}{dy}\right)-\mathrm{Da}_{\mathrm{e}}qa=0, \tag{S60}$$

$$\frac{d^2a}{dy^2}+n\frac{d}{dy}\left(a\frac{d\phi}{dy}\right)-\mathrm{Da}_{\mathrm{A}}qa=0, \tag{S61}$$

$$\frac{d^2b}{dy^2}+\left(n-1\right)\frac{d}{dy}\left(b\frac{d\phi}{dy}\right)+\mathrm{Da}_{\mathrm{A}}qa=0, \tag{S62}$$

$$\frac{du}{dy}+u\frac{d\phi}{dy}=0, \tag{S63}$$

$$\frac{dv}{dy}-v\frac{d\phi}{dy}=0, \tag{S64}$$

$$\psi=-\frac{dq}{dy}(0)-(z-1)q_0(1-q_0)\frac{d\phi}{dy}(0), \tag{S65}$$

$$\psi=\Lambda e^{\alpha(\xi+\phi_0)}\left(1-q_0\left(1+e^{-(\xi+\phi_0)}\right)\right), \tag{S66}$$

where q_0 is the boundary condition for Q at the electrode surface, and the dimensionless cathodic current is defined as positive for convenience. The dimensionless Damköhler numbers, $\mathrm{Da_e}$ and $\mathrm{Da_A}$, are defined as

$$\mathrm{Da_e} = d_\mathrm{f}^2 \frac{kC_\mathrm{A}^0}{D_\mathrm{e}}, \qquad \mathrm{Da_A} = d_\mathrm{f}^2 \frac{kC_\mathrm{P}^0}{D_\mathrm{A}}. \tag{S67}$$

Poisson's equation in dimensionless form becomes

$$\Theta \frac{d^2\phi}{dy^2} = -\left(zp + (z-1)q + \gamma\left(na + (n-1)b\right) + \beta\left(u - v\right)\right), \tag{S68}$$

with

$$\Theta = \frac{\lambda_D}{d_\mathrm{f}}, \qquad \gamma = \frac{C_\mathrm{A}^0}{C_\mathrm{P}^0}, \qquad \beta = \frac{C_\mathrm{I}^0}{C_\mathrm{P}^0}, \tag{S69}$$

where λ_D is the Deybe length defined as

$$\lambda_D = \sqrt{\frac{RT\varepsilon_r\varepsilon_0}{F^2 C_\mathrm{I}^0}}. \tag{S70}$$

3.4.3 Analytical solution

The system depends on a total of six dimensionless governing parameters:

$$\mathrm{Da_e} = d_\mathrm{f}^2 \frac{kC_\mathrm{A}^0}{D_\mathrm{e}}, \qquad \mathrm{Da_A} = d_\mathrm{f}^2 \frac{kC_\mathrm{P}^0}{D_\mathrm{A}}, \qquad \Theta = \frac{\lambda_D}{d_\mathrm{f}}, \qquad \gamma = \frac{C_\mathrm{A}^0}{C_\mathrm{P}^0}, \qquad \beta = \frac{C_\mathrm{I}^0}{C_\mathrm{P}^0}, \qquad \Lambda = \frac{d_\mathrm{f} k_s^0}{D_\mathrm{e}}.$$

The dimensionless governing parameters can aid in identifying the *asymptotic* behavior of the system by examining the limits of large or small values. This approach enables us to derive an approximate analytical solution for both the concentration profiles and current.

Firstly, the relative permittivity of DMF is $\varepsilon_r = 38.4$ [23], indicating that at the concentrations of supporting electrolyte used in this study ($C_\mathrm{I}^0 = 0.5$ M), the Debye length is approximatly $\lambda_D = 0.4$ nm. For a 1 μm thick film, this is sufficient to guarantee $\lambda_D \ll d_\mathrm{f}$ and consequently $0 < \Theta \ll 1$, across the entire span of the film. Examining the situation within each pore, the average pore size in Zn(NDI)@FTO is approximately 16 Å [1], which is roughly four times larger than the Debye length. We are also working at timescales much longer than $\lambda_D^2/D_\pm \approx 16$ μs, which is the characteristic relaxation time in any given pore, considering a lower limit on the ionic diffusivities in the MOF as $D_\pm \approx 10^{-10}$ cm^2 s^{-1}. These conditions ensure that charge screening takes place effectively within each pore, and we can assume that electroneutrality holds throughout the film. For $0 < x < d_\mathrm{f}$, we obtain $\Theta \to 0$, and from Eq. S68 this implies a singularly perturbed problem [19, 20] with the leading order outer solution

$$zp + (z-1)q + \gamma\left(na + (n-1)b\right) + \beta\left(u - v\right) = 0. \tag{S71}$$

We begin with Eqs. S63 and S64:

$$\frac{1}{u}\frac{du}{dy} = -\frac{d\phi}{dy}, \tag{S72}$$

$$\frac{1}{v}\frac{dv}{dy} = \frac{d\phi}{dy}. \tag{S73}$$

Integration yields

$$\int_1^{u(y)} \frac{1}{\tilde{u}}\, d\tilde{u} = -\int_\infty^y \phi'(\theta)\, d\theta \qquad \Rightarrow \qquad \ln u = -\phi, \tag{S74}$$

$$\int_1^{v(y)} \frac{1}{\tilde{v}}\, d\tilde{v} = \int_\infty^y \phi'(\theta)\, d\theta \qquad \Rightarrow \qquad \ln v = \phi, \tag{S75}$$

where we used the condition as $y \to \infty$, $u = 1$, $v = 1$, and $\phi = 0$ to evaluate the lower limit of integration. From which we can conclude

$$u(y) = e^{-\phi(y)}, \qquad v(y) = e^{\phi(y)}, \tag{S76}$$

yielding the classic equilibrium Boltzmann distribution for the mobile ions [10]. Furthermore, we have

$$u = v^{-1}. \tag{S77}$$

From the electroneutrality condition it follows that

$$\beta^{-1}[zp + (z-1)q] + \gamma\beta^{-1}[na + (n-1)b] + u - v = 0, \tag{S78}$$

with $\gamma\beta^{-1} = C_{\mathrm{A}}^0/C_{\mathrm{I}}^0$. In the present study, $C_{\mathrm{I}}^0 = 0.5$ M and $C_{\mathrm{A}}^0 \leq 0.036$ M, which puts an upper bound on the value for $\gamma\beta^{-1} \leq 0.072$. Additonally $\beta = \mathcal{O}(1)$, such that the dominate balance [19] of Eq. S78 gives

$$\gamma \ll \beta \sim 1, \tag{S79}$$

and the term $[na + (n-1)b]$ is negligible. Conservation of the redox active species implies $p + q = 1$. Combining these with Eq. S78 allows us to write

$$z - q = \beta(v - u). \tag{S80}$$

Differentiating both sides of Eq. S80, we find

$$-\frac{dq}{dy} = \beta\left(\frac{dv}{dy} - \frac{du}{dy}\right). \tag{S81}$$

Substituting in Eqs. S63 and S64 finally gives

$$-\frac{1}{\beta(u+v)}\frac{dq}{dy} = \frac{d\phi}{dy}. \tag{S82}$$

Furthermore, a combination of Eqs. S77 and S80 results in a polynomial for u:

$$u^2 + \beta^{-1}(q-z)u - 1 = 0. \tag{S83}$$

The relevant root of which is

$$u = \frac{q - z + \sqrt{(q-z)^2 + 4\beta^2}}{2\beta}. \tag{S84}$$

Selecting the positive root ensures u is real and positive for $q \geq 0$. Eqs. S77 and S80 further imply that

$$u + v = 2u + \frac{z-q}{\beta}. \tag{S85}$$

Using Eqs. S82, S84, and S85, we can now write the electric potential gradient as a function of q:

$$\frac{d\phi}{dy} = -\frac{1}{\sqrt{(q-z)^2 + 4\beta^2}}\frac{dq}{dy}. \tag{S86}$$

Substituting this into the governing Eqs. S60, S61, and the current expression leaves

$$\frac{d^2q}{dy^2} - (z-1)\frac{d}{dy}\left(\frac{q(1-q)}{\sqrt{(q-z)^2 + 4\beta^2}}\frac{dq}{dy}\right) - \mathrm{Da_e}qa = 0, \tag{S87}$$

$$\frac{d^2a}{dy^2} - n\frac{d}{dy}\left(\frac{a}{\sqrt{(q-z)^2 + 4\beta^2}}\frac{dq}{dy}\right) - \mathrm{Da_A}qa = 0, \tag{S88}$$

$$\psi = \left(\frac{(z-1)q_0(1-q_0)}{\sqrt{(q_0-z)^2 + 4\beta^2}} - 1\right)\frac{dq}{dy}(0). \tag{S89}$$

Integration of Eq. S86 results in an expression for the electrostatic potential as a function of $q(y)$:

$$\phi(y) = \ln\left(\frac{z - q(y) + \sqrt{(q(y)-z)^2 + 4\beta^2}}{z + \sqrt{z^2 + 4\beta^2}}\right). \tag{S90}$$

We can solve this resulting system of equations using the same perturbative approach as in the previous section, considering that the catalytic reaction is fast compared to the diffusion timescale of both the freely diffusing acceptor molecule and electron-hopping. As before, let $\epsilon_1 := \mathrm{Da}_\mathrm{e}^{-1}$ and $\epsilon_2 := \mathrm{Da}_\mathrm{A}^{-1}$ such that $0 < \epsilon_1, \epsilon_2 \ll 1$. We obtain the same leading order solution for the right and left outer layers:

$$a^L_{0,0} = 0, \quad q^R_{0,0} = 0. \tag{S91}$$

Subtracting Eq. S88 from Eq. S87 gives

$$\frac{d^2q}{dy^2} - \eta\frac{d^2a}{dy^2} - \frac{d}{dy}\left(\frac{(z-1)q(1-q) - \eta na}{\sqrt{(q-z)^2 + 4\beta^2}}\frac{dq}{dy}\right) = 0, \tag{S92}$$

where η is defined as before

$$\eta = \frac{\epsilon_2}{\epsilon_1} = \frac{D_\mathrm{A}C^0_\mathrm{A}}{D_\mathrm{e}C^0_\mathrm{P}}. \tag{S93}$$

Integration yields

$$\left(1 - \frac{(z-1)q(1-q) - \eta na}{\sqrt{(q-z)^2 + 4\beta^2}}\right)\frac{dq}{dy} - \eta\frac{da}{dy} = c_1. \tag{S94}$$

Applying the approximation that $D_\mathrm{A}C^0_\mathrm{A} \ll D_\mathrm{e}C^0_\mathrm{P}$, or $\eta \to 0$, at leading order Eq. S94 becomes

$$\left(1 - \frac{(z-1)q(1-q)}{\sqrt{(q-z)^2 + 4\beta^2}}\right)\frac{dq}{dy} = c_1. \tag{S95}$$

From Eq. S89, it follows that

$$c_1 = -\psi, \tag{S96}$$

which is only a function of q_0, β, and z. After integration we find

$$\begin{aligned} &q + \frac{1}{2}(z-1)(q+3z-2)\sqrt{(q-z)^2+4\beta^2} \\ &\quad + (z-1)\left((z-1)z - 2\beta^2\right)\ln\left(\sqrt{(q-z)^2+4\beta^2} + q - z\right) = -\psi y + c_2. \end{aligned} \tag{S97}$$

We are interested in the outer solutions for Q: $q \sim q^L_{0,0}$, $q^R_{0,0}$, particularly the left-hand outer solution since this will allow us to compute the current response. Using the boundary conditions $q(0) = q_0$ and $q^R_{0,0} = 0$ as $y \to 1$ to solve for c_2, yields an implicit equation for the dimensionless concentration profile of Q, $q(y)$, of the form

$$f(q, z, \beta) = (1-y)\psi + h(z, \beta), \tag{S98}$$

with

$$\begin{aligned} f(q, z, \beta) = {} & q + \frac{1}{2}(z-1)(q+3z-2)\sqrt{(q-z)^2+4\beta^2} \\ & + (z-1)\left((z-1)z - 2\beta^2\right)\ln\left(\sqrt{(q-z)^2+4\beta^2} + q - z\right), \end{aligned} \tag{S99}$$

$$h(z,\beta) = \frac{1}{2}(z-1)(3z-2)\sqrt{z^2+4\beta^2} + (z-1)\left((z-1)z - 2\beta^2\right)\ln\left(\sqrt{z^2+4\beta^2} - z\right), \tag{S100}$$

and

$$\psi = f(q_0, z, \beta) - h(z, \beta). \tag{S101}$$

Combining the electrostatic potential profile (Eq. S90) evaluated at $y = 0$ with the expression for the electrode kinetics (Eq. S66) results in

$$\psi = \Lambda e^{\alpha\xi}\left(\frac{z - q_0 + \sqrt{(z-q_0)^2 + 4\beta^2}}{z + \sqrt{z^2+4\beta^2}}\right)^{\alpha}\left(1 - q_0 - \frac{q_0 e^{-\xi}\left(z + \sqrt{z^2+4\beta^2}\right)}{z - q_0 + \sqrt{(z-q_0)^2 + 4\beta^2}}\right). \tag{S102}$$

For a given value of ξ, equating Eq. S101 and Eq. S102 allows us to implicitly calculate q_0, and therefore ψ, finally yielding the current-potential response.

3.5 Asymptotic approximation for the plateau current

Let us now examine the analytical result for the current response expressed by Eqs. S98-S101 in the limit as $\beta \to 0$, which represents low ion concentrations, where the effects of migration and the electric field have the greatest impact on the current response. In the experiment system under investigation, the charge on the oxidized linkers is $z = 0$. Additionally, $q_0 = 1$ corresponds to the plateau current. Substituting these values into Eq. S101 gives

$$\psi_{\mathrm{pl}} = 1 - 2\beta + \frac{1}{2}\sqrt{1+4\beta^2} + 2\beta^2\ln\left(2\beta(\sqrt{1+4\beta^2}+1)\right). \tag{S103}$$

A lowest order expansion for $0 < \beta \ll 1$ yields

$$\psi_{\mathrm{pl}} = \frac{3}{2} + \mathcal{O}(\beta). \tag{S104}$$

Back in dimensional units we have

$$i_{\mathrm{pl}} = \frac{3}{2}FS\frac{C_{\mathrm{P}}^0 D_{\mathrm{e}}}{d_{\mathrm{f}}}. \tag{S105}$$

Comparing this result to Eq. S44 (derived without including migration), the current increases by a factor of 3/2. This occurs when the ion concentration dips below that of the redox-active linkers and migration contributes significantly to the transport of both electrons and ion within the film.

3.6 Computational details and model verification

Eqs. S98 - S102 constitute a complete *analytical solution* to a catalytic cross reaction in a planar 1D redox film between an immobilized redox species and a mobile redox acceptor, when the reactions are fast on the diffusional timescales and when the reaction layer is located next to the film-solution interface. This solution provides the current as a function of potential, taking into account electromigration and field effects for both electron-hopping and mobile counter ions. The concentration profiles and current are expressed as implicit functions, necessitating the use of a numerical root-finding algorithm. To that end, concentration profiles and simulated cyclic voltammograms were generated numerically by evaluating Eqs. S98 - S102 on a discretized spatial domain with equal grid spacing using MATAB's *fzero* function. It is important to note, however, that despite this computational step, evaluating the analytical result presented here

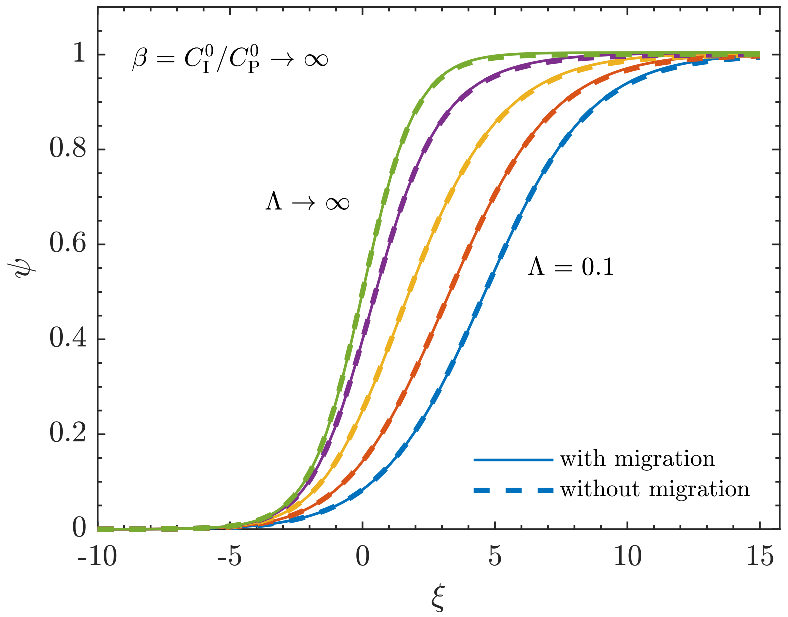

Figure S2. Simulated dimensionless cyclic voltammograms used to verify the analytical solution including migration given by Eqs. S101 - S102 when the mobile ion concentration is in excess, i.e., when $\beta = C_\mathrm{I}^0/C_\mathrm{P}^0 \to \infty$. The solid lines correspond to dimensionless voltammograms computed with Eqs. S101 - S102 at different values of the standard rate constant for interfacial electron transfer given by $\Lambda = d_\mathrm{f} k_s^0/D_\mathrm{e}$, while the dotted lines are calculated with Eq. S42 using the same parameter values: $\Lambda = 0.1$ (blue), 0.2 (red), 0.5 (orange), 2 (purple), 1×10^3 (green). Other parameters used: $\alpha = 0.5$, $\beta = 20$, with $\mathrm{Da_e} = 1 \times 10^4$, and $\mathrm{Da_A} = 1 \times 10^6$, which gives $\eta = \mathrm{Da_e}/\mathrm{Da_A} = 0.01$. Dimensionless current and potential are defined as $\psi = i/FSC_\mathrm{P}^0\left(D_\mathrm{e}/d_\mathrm{f}\right)$ and $\xi = -(F/RT)\left(E - E^0\right)$ respectively.

is considerably less demanding than alternative approaches. For instance, solving the full non-linear boundary value problem comprised of the system of differential equations (Eqs. S45 - S51) by employing finite-differnence method [24] would be computationally more intensive.

Verification of the analytical solution encompassing electromigration, as expressed by Eqs. S98 - S102, involved setting $\beta = C_\mathrm{I}^0/C_\mathrm{P}^0$ to a large value (see Figure S2). This choice effectively transforms the system into one resembling a fully-supported electrolyte, wherein the concentration of mobile counter ions is in excess compared to that of the analyte, and as a result there is not a significant electrostatic potential gradient across the film. In this context, the redox-active analyte corresponds to the NDI linkers within the MOF film. Under these conditions, our analytical solution aligns with the corresponding model in the absence migration, as represented by Eq. S42 in the preceding section.

4 Model validation with experimental results and DFT calculations

There are two key conditions used in the analytical model to obtain the current-potential expression. In this section, we will use experimental results combined with density functional theory (DFT) calculations to justify these two assumptions for the experimental system under investigation and validate the model.

Condition 1. The thin reaction layer is positioned next to the film-solution interface, meaning electrons must diffuse further than the acceptor:

$$\eta \ll 1, \text{ or equivalently } D_\mathrm{A} C_\mathrm{A}^0 \ll D_\mathrm{e} C_\mathrm{P}^0; \tag{S106}$$

Condition 2. The electron transfer reaction between the linkers and acceptor molecule is fast relative to the diffusion timescales for both electrons and acceptor:

$$\mathrm{Da_e} \gg 1 \text{ and } \mathrm{Da_A} \gg 1, \text{ or equivalently } kC_\mathrm{A}^0 \gg \frac{D_\mathrm{e}}{d_\mathrm{f}^2} \text{ and } kC_\mathrm{P}^0 \gg \frac{D_\mathrm{A}}{d_\mathrm{f}^2}. \tag{S107}$$

These conditions generate concentration profiles that mimic those obtained by an IDA electrode (Figure S1, right).

4.1 Experimental determination of mobile acceptor diffusivity

To show Condition 1 is valid (Eq. S106), we can use the experimental CVs to determine the intra-MOF diffusivity D_A of the mobile acceptor $[\mathrm{Co(bpy)_3}]^{3+}$. Consider the first wave (−0.12 V) in the presence of 36 mM $[\mathrm{Co(bpy)_3}]^{3+}$ (Figure S3a). In this potential range the MOF is in its neutral state, so the $[\mathrm{Co(bpy)_3}]^{3+}$ species is essentially diffusing through an inert film to the underlying electrode surface where electron transfer takes place. When the scan rate is decreased, however, a quasi-plateau emerges (Figure S3b). A steady-state voltammetric response is possible for a redox-active species diffusing through an electroinactive layer [25]. This occurs when the diffusion layer thickness of the mobile redox species reaches the size of the inert film and if the diffusion coefficient within the film is significantly less than in the bulk solution. Under these conditions the plateau current is given by

$$i_\mathrm{pl} = \frac{FSC_\mathrm{A}^0 D_\mathrm{A}}{d_\mathrm{f}}, \tag{S108}$$

where S is the geometric surface area of the film, C_A^0 is the bulk concentration of $[\mathrm{Co(bpy)_3}]^{3+}$, and D_A is the diffusion coefficient of the acceptor ($[\mathrm{Co(bpy)_3}]^{3+}$) within the film. Using this analysis, D_A was estimated to be 1.7×10^{-9} cm^2 s^{-1}, which is indeed much smaller than typical diffusion coefficients for small molecules in solution (10^{-5} cm^2 s^{-1}).

Given the experimentally determined magnitudes of D_e (10^{-10} cm^2 s^{-1}) and D_A (10^{-9} cm^2 s^{-1}), as well as the relative ratio of linker concentration to acceptor concentration, we find $D_\mathrm{A} C_\mathrm{A}^0 / D_\mathrm{e} C_\mathrm{P}^0 = 0.1 \ll 1$. This result fulfills the first condition (Eq. S106).

4.2 Estimation of the cross-exchange rate constant

Regarding the fast rate of electron transfer between linker and acceptor (Condition 2), we could corroborate this by utilizing the experimentally determined values of the diffusion coefficients to estimate a lower bound on the cross-exchange rate constant k for electron transfer between the $\mathrm{NDI}^{\bullet-}$ linkers and $[\mathrm{Co(bpy)_3}]^{3+}$. In the next sections, we use the experimental data and calculated Damköhler numbers to estimate a lower bound for k, such that Condition 2 is met. We then apply Marcus theory for outer sphere electron transfer in conjuction with DFT calculations to obtain an approximate value for k. This is compared to the lower bound from the experimental data to ensure that the model is internally consistent and Eq. S107 above is fulfilled with the experimental system at hand.

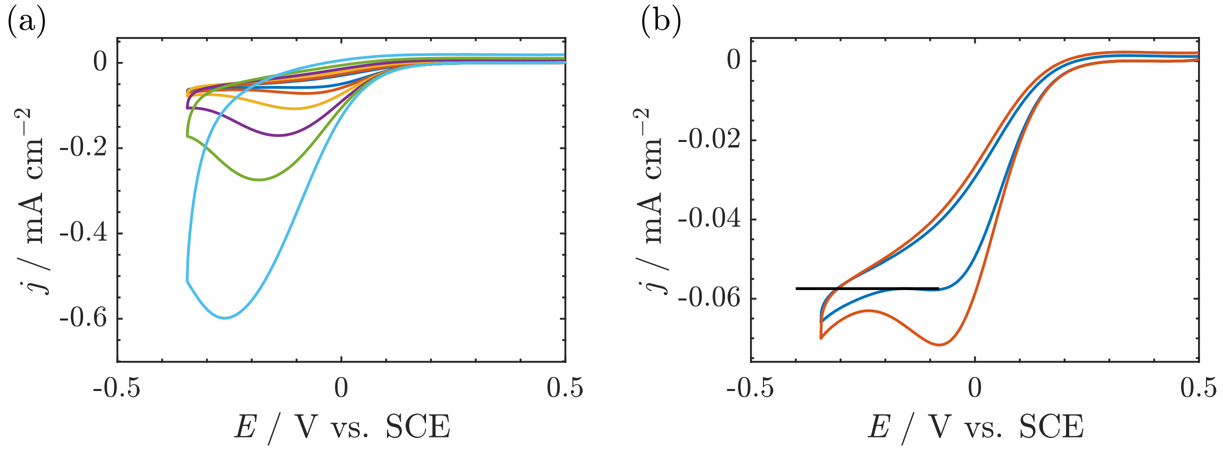

Figure S3. (a) CVs of Zn(NDI)@FTO with 36 mM $[Co(bpy)_3]^{3+}$ in 0.5 M $LiClO_4$ / DMF at $\nu =$ 10 mV s^{-1}, blue; 20 mV s^{-1}, red; 50 mV s^{-1}, orange; 100 mV s^{-1}, purple; 200 mV s^{-1}, green; 500 mV s^{-1}, cyan. (b) 36 mM $[Co(bpy)_3]^{3+}$ in 0.5 M $LiClO_4$ / DMF at scan rates of 10 mV s^{-1} (blue) and 20 mV s^{-1} (red); black line shows the quasi-plateau current (i_{pl}). The diffusion coefficient of $[Co(bpy)_3]^{3+}$ in the MOF film, D_{A}, was calculated using $i_{\mathrm{pl}} = FSC^0_{\mathrm{A}}D_{\mathrm{A}}/d_{\mathrm{f}}$.

4.2.1 Determining a lower bound for k using Damköhler numbers

From the experimentally determined values for D_{A} and D_{e}, we can find a lower bound on the bimolecular electron cross-exchange rate constant k. Fast reactions entails $\mathrm{Da_e}$, $\mathrm{Da_A} \gg 1$ (see also main text, Figure 3d). From this we can estimate the relative size of the two Damköhler numbers, $\eta = \mathrm{Da_e}/\mathrm{Da_A}$. With $D_{\mathrm{e}} = 5.5 \times 10^{-10}$ cm^2 s^{-1}, $D_{\mathrm{A}} = 1.7 \times 10^{-9}$ cm^2 s^{-1}, $C^0_{\mathrm{A}} = 36$ mM, and $C^0_{\mathrm{P}} = 1$ M, we find $\eta = 0.1$, such that $\mathrm{Da_e} \ll \mathrm{Da_A}$. Ensuring $\mathrm{Da_e}$ is at least larger than 10^2, corresponding to fast reactions on the diffusional timescale, it follows that

$$\mathrm{Da_e} = d_{\mathrm{f}}^2 \frac{kC^0_{\mathrm{A}}}{D_{\mathrm{e}}} = (10^{-4}\ \mathrm{cm})^2 \frac{k \cdot 3.6 \times 10^{-2}\ \mathrm{M}}{5.5 \times 10^{-10}\ \mathrm{cm^2\ s^{-1}}} \geq 10^2 \ \Rightarrow\ k \geq 1.5 \times 10^2\ \mathrm{M^{-1}\ s^{-1}},$$

$$\mathrm{Da_A} = d_{\mathrm{f}}^2 \frac{kC^0_{\mathrm{P}}}{D_{\mathrm{A}}} = (10^{-4}\ \mathrm{cm})^2 \frac{k \cdot 1\ \mathrm{M}}{1.7 \times 10^{-9}\ \mathrm{cm^2\ s^{-1}}} \geq 10^3 \ \Rightarrow\ k \geq 1.7 \times 10^2\ \mathrm{M^{-1}\ s^{-1}}.$$

This means we can estimate a lower bound on k to a first order approximation as

$$k \geq 10^2\ \mathrm{M^{-1}\ s^{-1}}, \tag{S109}$$

which is likely obtained experimentally for a cross reaction with sufficient driving force. In the next section, we calculate an approximate value for k to confirm that this lower bound is satisfied by the experimental system.

4.2.2 Marcus theory and DFT calculations

We used Marcus theory to estimate the rate of outersphere electron cross exchange between the reduced NDI linkers and $[Co(bpy)_3]^{3+}$ [26, 27]. In the non-adiabatic limit, the electron-transfer rate constant k can be found by the Marcus cross relation [28], which is given by

$$k = \sqrt{k_{11}k_{22}K_{12}f_{12}}, \tag{S110}$$

where k_{11} and k_{22} are the individual self-exchange rate constants for NDI/NDI$^{\bullet-}$ and Co(III)/Co(II) respectively. The equilibrium constant for the overall cross-exchange reaction, K_{12}, is

$$K_{12} = \exp\left(\frac{-\Delta G^0}{RT}\right), \tag{S111}$$

where ΔG^0 is the standard free energy for the reaction (computed from the experimentally determined standard potentials of the NDI and $[Co(bpy)_3]^{3+}$ couples; $\Delta E^0 = 0.8$ V), and f_{12} is a known function of the self-exchange rate constants. Here, for simplicity, we take f_{12} to be close to unity, as is often the case, and assume cancellation of the work terms as a first order approximation [28].

For the NDI linkers, the bimolecular self-exchange rate constant can be estimated using the electron-hopping diffusion coefficient and the relation

$$k_{11} = \frac{6D_\mathrm{e}}{C^0_\mathrm{P}\delta^2}, \tag{S112}$$

where δ is the center-to-center distance between pairs of linkers [6, 29] (taken as twice the radius of the NDI core $\delta = 7$ Å, obtained from the DFT optimized structures; see below for details). The self-exchange rate constant for Co(III)/Co(II) is given by

$$k_{22} = Z \exp\left(\frac{\lambda}{4RT}\right), \tag{S113}$$

where λ is the reorganization energy, and Z is the collision frequency [30] (10^{10} M^{-1} s^{-1}).

The reorganization energy can be broken down into two components $\lambda = \lambda_\mathrm{int} + \lambda_\mathrm{out}$: internal rearrangement reflecting structural differences between the reactant and product states λ_int, and a contribution from the solvent λ_out. These are defined as

$$\lambda_\mathrm{int} = [E_p(g_r) - E_p(g_p)] + [E_r(g_p) - E_r(g_r)], \tag{S114}$$

where $E_r(g_p)$ is the energy of the reactants with the nuclear geometry of the products and $E_p(g_r)$ is the energy of the products in the nuclear geometry of the reactants [27]. $E_r(g_r)$ and $E_p(g_p)$ are the typical ground state energies of the reactants and products respectively. The outer reorganization energy is

$$\lambda_\mathrm{out} = (\Delta e)^2 \left(\frac{1}{n^2} - \frac{1}{\varepsilon_r}\right)\left(\frac{1}{2r_\mathrm{D}} + \frac{1}{2r_\mathrm{A}} - \frac{1}{r_\mathrm{AD}}\right), \tag{S115}$$

where Δe is the total charge transferred ($\Delta e = 3.8 \times 10^{-5}$ m eV [30]), n and ε_s are the refractive index and relative permittivity respectively (in DMF $\varepsilon_r = 38.4$ and $n = 1.43$), r_D is the radius of the donor, r_A is the radius of the acceptor, and r_AD is the donor acceptor distance [28]. Distances for $[Co(bpy)_3]^{3+}/[Co(bpy)_3]^{2+}$ self-exchange were obtained from the DFT optimized structures (see below for details) as $r_\mathrm{A} = r_\mathrm{D} = 5.5$ Å and $r_\mathrm{DA} = 2r_\mathrm{A} = 2r_\mathrm{D}$. The energies of the reactant and product states were calculated using DFT and are tabulated below.

Taken together, these calculations yield

$$k = 6 \times 10^7 \ \mathrm{M}^{-1}\ \mathrm{s}^{-1}. \tag{S116}$$

This value exceeds the lower bound for electron cross exchange predicted by the experimentally determined Damköhler numbers ($k \geq 10^2$ M^{-1} s^{-1}), fulfills Condition 2 (Eq. S107) and shows the analysis to be internally consistent.

We note that due to geometric localization at an interface, the value of the collision frequency has been calculated to be significantly less than the diffusion limit in solution (10^{10} M^{-1} s^{-1}), resulting from a reduction of dimensionality [31]. In which case, diffusion-controlled rate constants as low as $10^5 - 10^4$ M^{-1} s^{-1} may be observed. Even if this is the case in the interior of the MOF film, we obtain $k = 6 \times 10^4$ M^{-1} s^{-1}, which is still sufficiently fast to ensure that the assumptions of the model are fulfilled in the experimental system.

4.2.3 DFT computational details

DFT calculations were performed using the Gaussian 16 Rev. C.02 software [32]. The B3LYP hybrid functional [33–36] with Grimme's D3 dispersion corrections [37, 38] and a Def2TZVP basis set [39, 40] with ultrafine grid and very tight convergence criteria were utilized for geometry optimizations. The COnductor-like Screening MOdel (COSMO) [41, 42] was used to include solvation effects from the experimental solvent of choice N,N-Dimethylformamide (DMF). Geometry optimizations were first performed on the molecules NDI, $NDI^{\bullet -}$, $[Co(bpy)_3]^{3+}$ and $[Co(bpy)_3]^{2+}$. In order to calculate the internal reorganization energies according to Eq. S114, further single point energy calculations were carried out on the aforementioned optimized structures, albeit with a different charge, to obtain the terms $E_p(g_r)$ and $E_r(g_p)$. Frequency calculations were performed on the optimized geometries of NDI, $NDI^{\bullet -}$, $[Co(bpy)_3]^{3+}$, and $[Co(bpy)_3]^{2+}$ to ensure that they reached an energy minimum, without any imaginary frequency. Thereafter, thermal corrections using the rigid rotor harmonic oscillator (RRHO) approximation were applied to the final optimized and single point energy geometries using their output files from vibrational frequency calculations via the SHERMO program that calculates various thermodynamic parameters of molecules [43], through the online SHERMO application (https://atomistica-online-shermo.anvil.app/) of the atomistica.online [44]. The following settings were applied:

**General settings**
Low frequency treatment: Harmonic
Evaluation mode: All terms (incl. translation and rotation) were used.
Conc. change [atm]: 0
Point group: Automatic
Atomic masses: Same as output file
**Scanning settings**
Temperature: 298.15 K
Pressure: 1 atm.
**Scaling settings**
ZPE : 0.963
U(T) - U(0) : 1.0
S(T) : 1.0
Heat capacity : 1.0

A zero point energy (ZPE) scaling factor of 0.963 was used (the value is listed in NIST database for the B3LYP ultrafine functional and TZVP basis set: https://cccbdb.nist.gov/vsfx.asp).

$[Co(bpy)_3]^{3+}$ is reported to exist in low-spin state while $[Co(bpy)_3]^{2+}$ is debated to exist in both high spin and low spin states. According to a recent report, $[Co(bpy)_3]^{2+}$ in aqueous solution has approximately ∼40% low-spin and ∼60% high-spin state components, determined by a combination of computations and experiments [45]. Our DFT calculations reveal the high spin state to be 4.98 kcal mol^{-1} lower in energy than the low spin state in DMF solvent, post thermal corrections using the parameters mentioned above. Therefore we chose the high spin $[Co(bpy)_3]^{2+}$ for this calculation. The standard potential of the Co(III) (low spin) / Co(II) (high spin) redox couple was calculated by DFT to be +0.30 V versus SCE in DMF solvent, a value only 30 mV higher than the experimentally obtained value of +0.27 V versus SCE in

DMF using $LiClO_4$ electrolyte at 298 K. The internal reorganization energies (λ_{int}) of the donor and the acceptor molecules as per Eq. S114 are tabulated below. All the energies (E) listed are obtained after employing thermal corrections using the SHERMO code.

	E ($[Co(bpy)_3]^n$ / eV)	E (NDI^m / eV)
$E_p(g_r)$	−78070.0247177241	−42320.0836740960
$E_p(g_p)$	−78071.6406666537	−42320.3370802492
$E_r(g_p)$	−78065.6248141010	−42324.0447721411
$E_r(g_r)$	−78066.6838899135	−42324.2584333249
λ_{int}	2.675	0.467

5 Summary of parameters and symbols

5.1 Dimensional parameters (Table S1)

parameter	symbol	value (units)
film thickness	d_{f}	$\sim 1\,\mu$m
electron-hopping diffusion coefficient (steady-state)	D_{e}	5.5×10^{-10} cm^2 s^{-1}
electron-hopping diffusion coefficient (transient)	$D_{\mathrm{e}}^{\mathrm{app}}$	2.5×10^{-9} cm^2 s^{-1}
acceptor (susbstrate) diffusion coefficient	D_{A}	1.7×10^{-9} cm^2 s^{-1}
bulk acceptor concentration (Co^{3+})	C_{A}^{0}	36 mM
total redox-active linker concentration (NDI)	C_{P}^{0}	1 M
mobile cation/anion bulk concentration	C_{I}^{0}	0.5 M
charge on linker (oxidized form)	z	0
charge on acceptor (oxidized form)	n	+3
standard rate constant	k_s^0	1×10^{-5} cm s^{-1}
transfer coefficient	α	0.5
diffusion current density	$i_{\mathrm{D}}/S = FC_{\mathrm{P}}^{0}D_{\mathrm{e}}/d_{\mathrm{f}}$	0.55 mA cm^{-2}
cross exchange rate constant for $\mathrm{NDI}^{\bullet-} + \mathrm{Co}^{3+} \rightarrow \mathrm{NDI} + \mathrm{Co}^{2+}$	k	$\geq 10^2$ M^{-1} s^{-1}

Table S1: Summary of dimensional parameters and their corresponding measured values used to simulate concentration profiles and current response.

5.2 Governing dimensionless parameters (Table S2)

parameter	symbol	meaning
electron-hopping Damköhler number	$\mathrm{Da_e}$	$\frac{\text{rate of catalytic rxn}}{\text{rate of electron diffusion}}$ $\frac{\text{film thickness}}{\text{diffusion layer thickness}\,(e^-)}$
acceptor Damköhler number	$\mathrm{Da_A}$	$\frac{\text{rate of catalytic rxn}}{\text{rate of acceptor diffusion}}$ $\frac{\text{film thickness}}{\text{diffusion layer thickness (acceptor)}}$
surface ET Damköhler number	Λ	$\frac{\text{rate of interfacial ET}}{\text{rate of electron diffusion}}$
diffusion competition parameter†	η	$\frac{\text{rate of acceptor diffusion}}{\text{rate of electron diffusion}}$ $\frac{\text{diffusion layer thickness (acceptor)}}{\text{diffusion layer thickness}\,(e^-)}$
dimensionless Debye length	Θ	$\frac{\text{Debye length}}{\text{film thickness}}$
acceptor excess parameter	γ	$\frac{\text{bulk acceptor concentration}}{\text{redox-active linker concentration}}$
mobile ion excess parameter	β	$\frac{\text{bulk supporting ion concentration}}{\text{redox-active linker concentration}}$

symbol	definition	value
$\mathrm{Da_e}$	$d_\mathrm{f}^2 \frac{kC_\mathrm{A}^0}{D_\mathrm{e}}$	$\gg 1$
$\mathrm{Da_A}$	$d_\mathrm{f}^2 \frac{kC_\mathrm{P}^0}{D_\mathrm{A}}$	$\gg 1$
Λ	$\frac{d_\mathrm{f} k_s^0}{D_\mathrm{e}}$	1.3
η	$\frac{D_\mathrm{A} C_\mathrm{A}^0}{D_\mathrm{e} C_\mathrm{P}^0}$	0.1
Θ	$\frac{\lambda_D}{d_\mathrm{f}}$	4×10^{-4}
γ	$\frac{C_\mathrm{A}^0}{C_\mathrm{P}^0}$	0.036
β	$\frac{C_\mathrm{I}^0}{C_\mathrm{P}^0}$	0.5

Table S2: Summary of dimensionless parameters, their corresponding physical meaning (ET = electron transfer), and determined values. †In fact, η is a derived parameter and is not independent from the other dimensionless groups. It is simply the ratio of the two Damköhler numbers $\eta = \mathrm{Da_e}/\mathrm{Da_A}$.

5.3 Summary of analytical results

Here we summarize the main results from the physico-mathematical model. The dimensionless analytical expressions for the concentration profiles of each species as well as the current-potential response, both in the absence and presence of electromigration, are listed below.

5.3.1 Diffusion only

Current:

$$\psi = \frac{1}{1 + \frac{1}{\Lambda} e^{-\alpha\xi} + e^{-\xi}} + \frac{\eta\left(1 + e^{-\xi}\right)}{1 + \frac{1}{\Lambda} e^{-\alpha\xi} + e^{-\xi}}.$$

Concentration profiles:

$$q = -\left(\eta + \psi\right) y - \left(\eta - \psi\right), \qquad a = \frac{\psi}{\eta}\left(y - 1\right) + 1.$$

5.3.2 Including electromigration

Common functions:

$$\begin{aligned} f(q, z, \beta) = & \, q + \frac{1}{2}(z-1)(q + 3z - 2)\sqrt{(q-z)^2 + 4\beta^2} \\ & + (z-1)\left((z-1)z - 2\beta^2\right) \ln\left(\sqrt{(q-z)^2 + 4\beta^2} + q - z\right), \end{aligned}$$

$$\begin{aligned} h(z, \beta) = & \, \frac{1}{2}(z-1)(3z-2)\sqrt{z^2 + 4\beta^2} \\ & + (z-1)\left((z-1)z - 2\beta^2\right) \ln\left(\sqrt{z^2 + 4\beta^2} - z\right). \end{aligned}$$

Current:

$$\psi = f(q_0, z, \beta) - h(z, \beta),$$

$$\psi = \Lambda e^{\alpha\xi}\left(\frac{z - q_0 + \sqrt{(z-q_0)^2 + 4\beta^2}}{z + \sqrt{z^2 + 4\beta^2}}\right)^{\alpha}\left(1 - q_0 - \frac{q_0 e^{-\xi}\left(z + \sqrt{z^2 + 4\beta^2}\right)}{z - q_0 + \sqrt{\left(z - q_0\right)^2 + 4\beta^2}}\right).$$

Concentration profile of Q:

$$f(q(y), z, \beta) = (1-y)\psi + h(z, \beta).$$

Electric potential:

$$\phi(y) = \ln\left(\frac{z - q(y) + \sqrt{(q(y) - z)^2 + 4\beta^2}}{z + \sqrt{z^2 + 4\beta^2}}\right).$$

Concentration profiles of mobile ions:

$$u(y) = e^{-\phi(y)}, \qquad v(y) = e^{\phi(y)}.$$

Plateu current (for $z = 0$):

$$\psi_{\mathrm{pl}} = 1 - 2\beta + \frac{1}{2}\sqrt{1 + 4\beta^2} + 2\beta^2 \ln\left(2\beta(\sqrt{1 + 4\beta^2} + 1)\right).$$

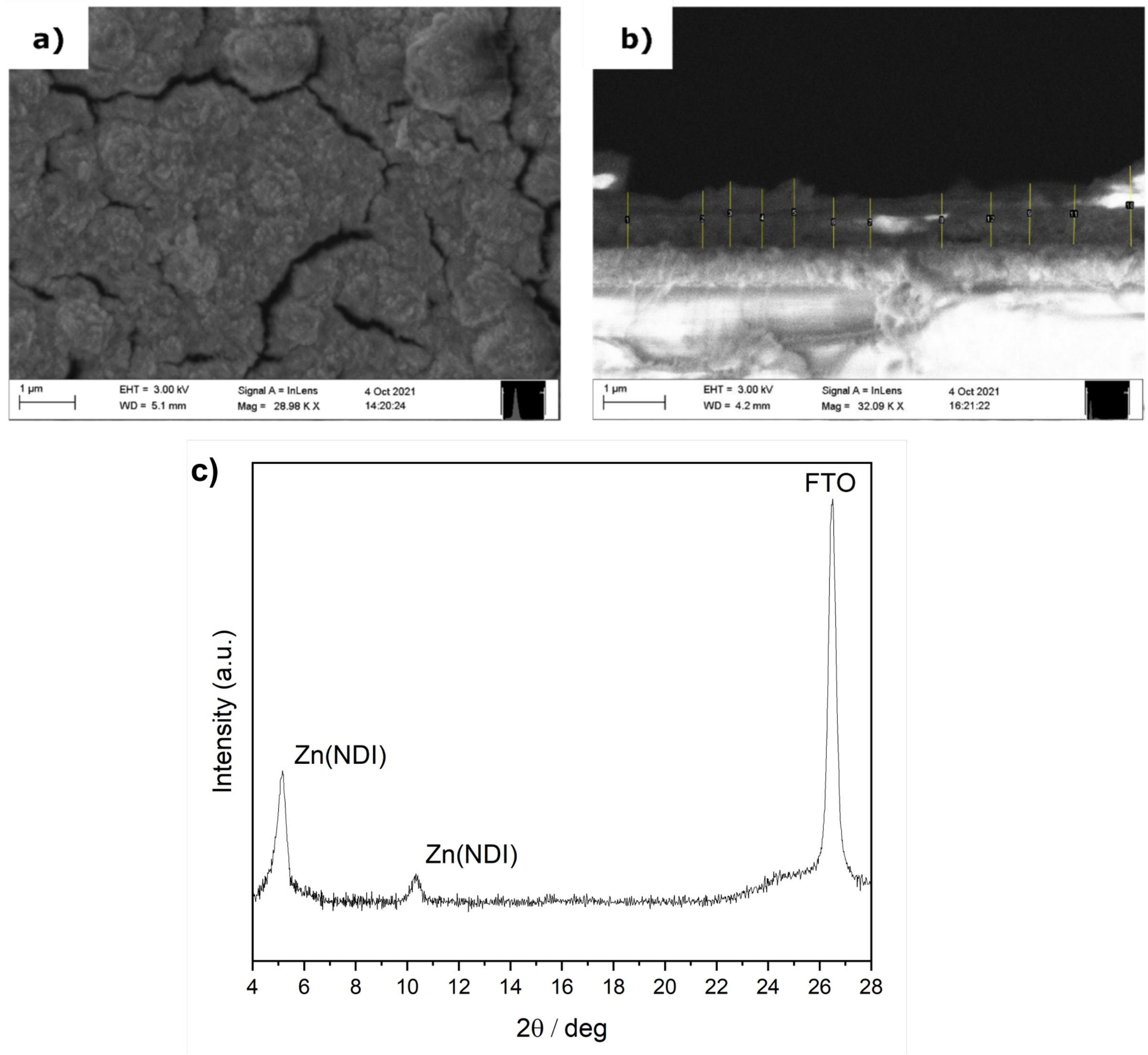

Figure S4. SEM images of Zn(NDI)@FTO (a) top down, and (b) cross section. Film thickness determined from cross sections by measuring thickness at multiple points (yellow lines) with ImageJ [3] and averaging all values for $d_f \approx 1$ μm. (c) PXRD of Zn(NDI) thin films. Peaks at $2\theta = 5.1°$ and $10.3°$ match reported values from thin films and bulk powder samples [1]. The large peak at $26.5°$ arises from the FTO substrate.

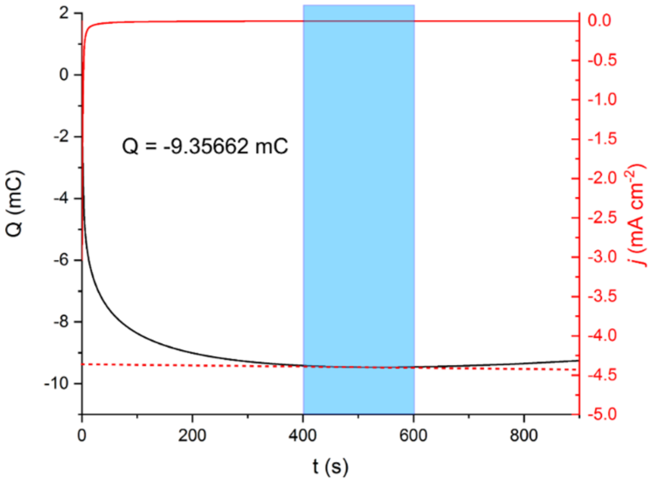

Figure S5. Chronoamperometry of Zn(NDI)@FTO ($d_f \sim 1\mu$m) showing current density (red) and charge passed (black) recorded after stepping the potential from −0.25 V to −0.85 V vs. SCE in DMF with 0.5 M $LiClO_4$ as the supporting electrolyte. Linear fit (red dashed line) of flat region of charge plot shows charge passed for complete reduction of film. The electroactive surface concentration of NDI was calculated according to $\Gamma_e = |Q|/(FS)$, where Γ_e is the electroactive linker concentration (mol cm^{-2}), Q is the charge (C), F is Faraday's constant (C mol^{-1}), and S is the surface area of the MOF-modified electrode (cm^2). This resulted in $\Gamma_e = 9.0 \times 10^{-8}$ mol cm^{-2}. With a film thickness of 1 μm, the total volumetric concentration of linker is approximately 0.9 M. This matched well with the value obtained from the charged passed under the cathodic wave of CVs at slow scan rates (see Figure 2b in the main text).

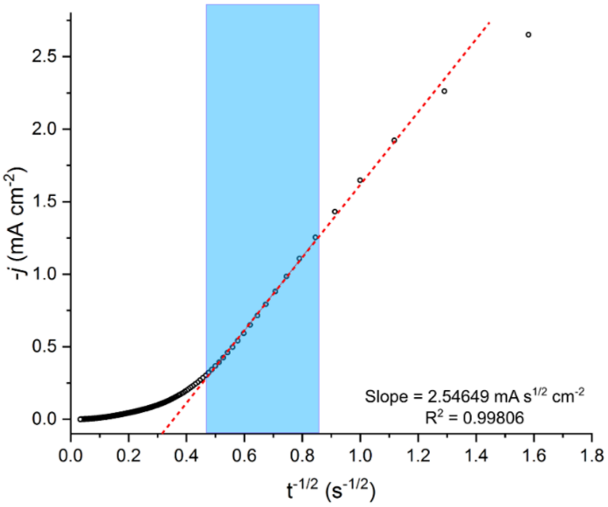

Figure S6. Cottrell plot of Zn(NDI)@FTO from chronoamperometry experiment shown in Figure S4, recorded after stepping the potential from −0.25 V to −0.85 V vs. SCE in 0.5 M $LiClO_4$ / DMF. Linear fit is displayed in red with blue box indicating the time points considered for linear fit. Time step = 0.2 s. The transient apparent diffusion coefficient D_e^{app} for electron hopping is extracted from the slope using the Cottrell equation. With $\Gamma_e = 9.0 \times 10^{-8}$ mol cm^{-2}, this results in $D_e^{app} = 2.5 \times 10^{-9}$ cm^2 s^{-1}.

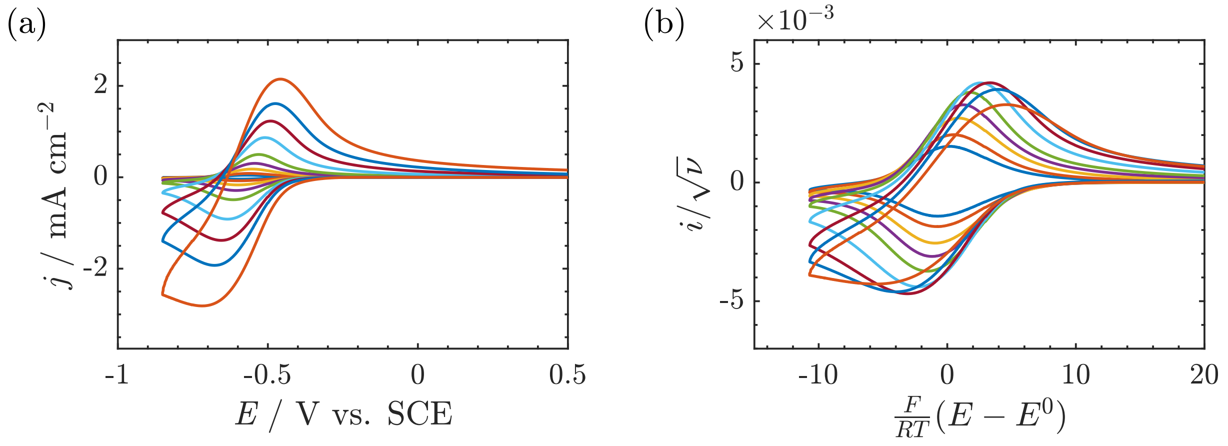

Figure S7. (a) Cyclic voltammograms of Zn(NDI)@FTO in 0.5 M $LiClO_4$/DMF at scan rates from 1 mV s^{-1} up to to 500 mV s^{-1}. (b) Normalized CVs under conditions same as in (a) with the current and potential axis normalized to the scan rate and the formal potential of the NDI/NDI$^{\bullet-}$ couple, respectively, showing proportionality of the peak current with $\sqrt{\nu}$ at higher scan rates.

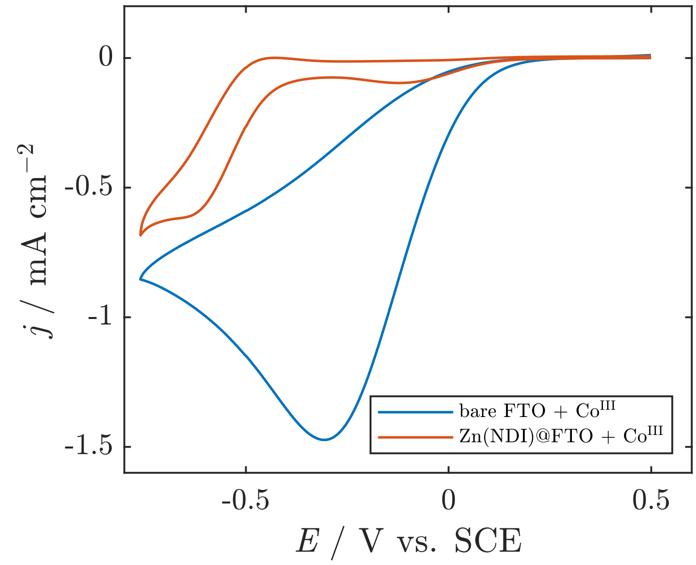

Figure S8. Background scan on bare FTO (blue) with comparison of current response of Zn(NDI)@FTO (red) in the presence of 36 mM $[Co(bpy)_3]^{3+}$ at 50 mV s^{-1} in DMF. The supporting electrolyte was 0.5 M $LiClO_4$.

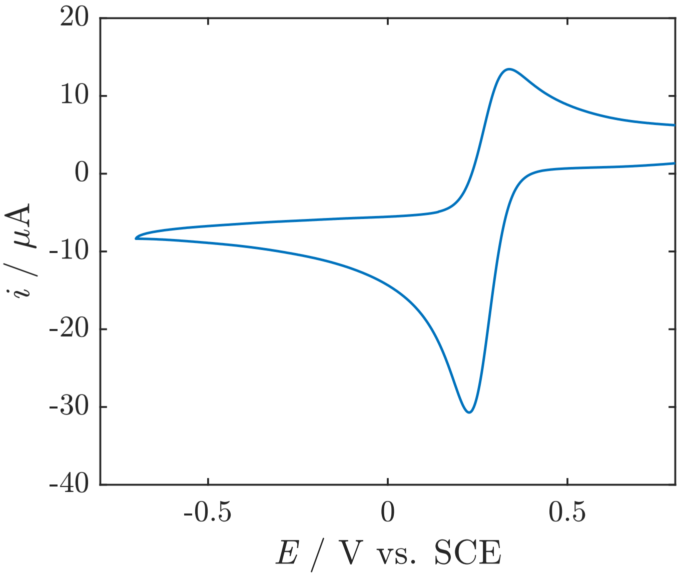

Figure S9. CV of 3 mM $[Co(bpy)_3]^{3+}$ in 0.5 M $LiClO_4$ / DMF using a 0.071 cm^2 glassy carbon disk working electrode at 100 mV s^{-1}. The Co(III)/Co(II) couple is displayed $E^0_{A/B}$= 0.27 V vs. SCE.

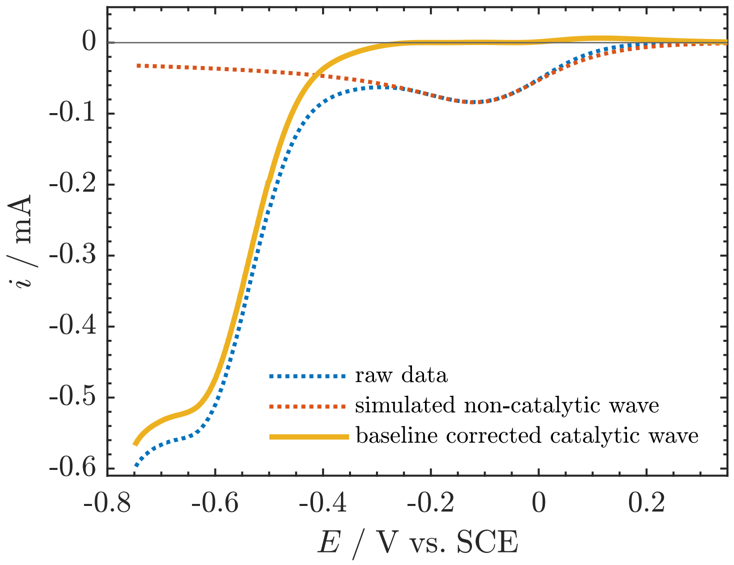

Figure S10. Background correction applied to steady-state catalytic CV in the presence of 36 mM $[Co(bpy)_3]^{3+}$ at 50 mV s^{-1} in DMF with 0.5 M $LiClO_4$. A simulated cyclic voltammogram was generated numerically using finite difference method (FDM) for the non-catalytic diffusion wave at −0.12 V (dotted red line; corresponding to the direct reduction of $[Co(bpy)_3]^{3+}$ at the underlying FTO electrode). A backwards implicit discretization scheme with second order central differencing for the second spatial derivative was used on an expanding spatial grid [24]. The current was computed using a forward two-point discretization for the first spatial derivative. Interfacial electron transfer was described by Butler–Erdey-Grúz–Volmer electrode kinetics [17, 18]. This simulated CV was subtracted from the raw data (blue dotted line) to remove residual non-catalytic current from the foot of the catalytic wave for quantitative analysis. The resulting baseline corrected voltammogram is presented (orange solid line). Backward scans were removed for clarity.

References

(1) Wade, C. R.; Li, M.; Dincă, M. *Angew. Chem. Int. Ed.* **2013**, *52*, 13377–13381.

(2) Wade, C. R.; Corrales-Sanchez, T.; Narayan, T. C.; Dincă, M. *Energy Environ. Sci.* **2013**, *6*, 2172–2177.

(3) Schneider, C. A.; Rasband, W. S.; Eliceiri, K. W. *Nature Methods* **2012**, *9*, 671–675.

(4) Savéant, J.-M. *J. Phys. Chem.* **1988**, *92*, 1011–1013.

(5) Savéant, J.-M. *J. Phys. Chem.* **1988**, *92*, 4526–4532.

(6) Anson, F. C.; Blauch, D. N.; Savéant, J.-M.; Shu, C. F. *J. Am. Chem. Soc.* **1991**, *113*, 1922–1932.

(7) Bediako, D. K.; Costentin, C.; Jones, E. C.; Nocera, D. G.; Savéant, J. M. *J. Am. Chem. Soc.* **2013**, *135*, 10492–10502.

(8) Ahn, S.; Son, M.; Singh, V.; Yun, A.; Baik, M.-H.; Byon, H. R. *J. Am. Chem. Soc.* **2024**, *146*, 4521–4531.

(9) Thiele, E. W. *Ind. Eng. Chem.* **1939**, *31*, 916–920.

(10) Newman, J.; Thomas-Alyea, K. E., *Electrochemical Systems*; Wiley: 2004.

(11) Chidsey, C. E. D.; Murray, R. W. *J. Phys. Chem.* **1986**, *90*, 1479–1484.

(12) Andrieux, C.; Savéant, J.-M. *J. Electroanal. Chem. Interfacial Electrochem.* **1980**, *111*, 377–381.

(13) Laviron, E. *J. Electroanal. Chem. Interfacial Electrochem.* **1980**, *112*, 1–9.

(14) Savéant, J.-M. *J. Electroanal. Chem. Interfacial Electrochem.* **1986**, *201*, 211–213.

(15) Costentin, C.; Savéant, J.-M. *ChemElectroChem* **2015**, *2*, 1774–1784.

(16) Andrieux, C. P.; Dumas-Bouchiat, J. M.; Savéant, J.-M. *J. Electroanal. Chem.* **1984**, *169*, 9–21.

(17) Erdey-Grúz, T.; Volmer, M. *Zeitschrift für Physikalische Chemie* **1930**, *150A*, 203–213.

(18) Butler, J. A. V. *Trans. Faraday Soc* **1924**, *19*, 734–739.

(19) Bender, C. M.; Orszag, S. A., *Advanced Mathematical Methods for Scientists and Engineers I: Asymptotic Methods and Perturbation Theory*, 1st ed.; Springer New York, NY: 1999.

(20) Holmes, M. H., *Introduction to Perturbation Methods*, 2nd ed.; Springer New York, NY.

(21) Amatore, C.; Pebay, C.; Scialdone, O.; Szunerits, S.; Thouin, L. *Chem. Eur. J.* **2001**, *7*, 2933–2939.

(22) Compton, R. G.; Laborda, E.; Ward, K. R., *Understanding Voltammetry: Simulation of Electrode Processes*; Imperial College Press: 2013.

(23) Hunger, J.; Buchner, R.; Kandil, M. E.; May, E. F.; Marsh Kenneth N. and Hefter, G. *J. Chem. Eng. Data* **2010**, *55*, 2055–2065.

(24) Britz, D.; Strutwolf, J., *Digital Simulation in Electrochemistry*, 4th ed.; Springer Cham: 2016.

(25) Menshykau, D.; Compton, R. G. *Langmuir* **2009**, *25*, 2519–2529.

(26) Van Wyk, A.; Smith, T.; Park, J.; Deria, P. *J. Am. Chem. Soc.* **2018**, *140*, 2756–2760.

(27) Patwardhan, S.; Schatz, G. C. *J. Phys. Chem. C* **2015**, *119*, 24238–24247.

(28) Marcus, R.; Sutin, N. *Biochim. Biophys. Acta* **1985**, *811*, 265–322.

(29) Costentin, C.; Savéant, J.-M., *Elements of Molecular and Biomolecular Electrochemistry*, 2nd ed.; John Wiley & Sons, Inc.: Hoboken, NJ, 2019.

(30) Zerk, T. J.; Saouma, C. T.; Mayer, J. M.; Tolman, W. B. *Inorganic Chemistry* **2019**, *58*, 14151–14158.

(31) Astumian, R. D.; Schelly, Z. A. *J. Am. Chem. Soc.* **1984**, *106*, 304–308.

(32) Frisch, M. J. et al. Gaussian 16 Revision C.02, Gaussian Inc. Wallingford CT, 2019.

(33) Becke, A. D. *J. Chem Phys.* **1993**, *98*, 5648–5652.

(34) Lee, C.; Yang, W.; Parr, R. G. *Phys. Rev. B* **1988**, *37*, 785–789.

(35) Vosko, S. H.; Wilk, L.; Nusair, M. *Can. J. Phys.* **1980**, *58*, 1200–1211.

(36) Stephens, P. J.; Devlin, F. J.; Chabalowski, C. F.; Frisch, M. J. *J. Phys. Chem.* **1994**, *98*, 11623–11627.

(37) Grimme, S.; Antony, J.; Ehrlich, S.; Krieg, H. *J. Chem. Phys.* **2010**, *132*, 154104.

(38) Bursch, M.; Mewes, J.-M.; Hansen, A.; Grimme, S. *Angew. Chem. Int. Ed.* **2022**, *61*, e202205735.

(39) Weigend, F.; Ahlrichs, R. *Phys. Chem. Chem. Phys.* **2005**, *7*, 3297–3305.

(40) Weigend, F. *Phys. Chem. Chem. Phys.* **2006**, *8*, 1057–1065.

(41) Klamt, A.; Schüürmann, G. *J. Chem. Soc., Perkin Trans. 2* **1993**, 799–805.

(42) Klamt, A., *From Quantum Chemistry to Fluid Phase Thermodynamics and Drug Design*; Elsevier: Boston, MA, 2005.

(43) Lu, T.; Chen, Q. *Comput. Theor. Chem.* **2021**, *1200*, 113249.

(44) Armaković, S.; Armaković, S. J. *Molecular Simulation* **2023**, *49*, 117–123.

(45) Sreekantan Nair Lalithambika, S.; Golnak, R.; Winter, B.; Atak, K. *Inorg. Chem.* **2019**, *58*, 4731–4740.
